# Inflammatory profile of eosinophils in asthma-COPD overlap and eosinophilic COPD: a multi-omics study

**DOI:** 10.3389/fimmu.2024.1445769

**Published:** 2024-10-08

**Authors:** Keeya Sunata, Jun Miyata, Yusuke Kawashima, Ryo Konno, Masaki Ishikawa, Yoshinori Hasegawa, Ryuta Onozato, Yo Otsu, Emiko Matsuyama, Hisashi Sasaki, Shinichi Okuzumi, Takao Mochimaru, Katsunori Masaki, Hiroki Kabata, Shotaro Chubachi, Makoto Arita, Koichi Fukunaga

**Affiliations:** ^1^ Division of Pulmonary Medicine, Department of Medicine, Keio University School of Medicine, Tokyo, Japan; ^2^ Laboratory for Metabolomics, RIKEN Center for Integrative Medical Sciences, Kanagawa, Japan; ^3^ Department of Applied Genomics, Kazusa DNA Research Institute, Chiba, Japan; ^4^ Division of Infectious Diseases and Respiratory Medicine, Department of Internal Medicine, National Defense Medical College, Saitama, Japan; ^5^ Department of Respiratory Medicine, National Hospital Organization Tokyo Medical Center, Tokyo, Japan; ^6^ Graduate School of Medical Life Science, Yokohama City University, Kanagawa, Japan; ^7^ Division of Physiological Chemistry and Metabolism, Graduate School of Pharmaceutical Sciences, Keio University, Tokyo, Japan; ^8^ Human Biology-Microbiome-Quantum Research Center (WPI-Bio2Q), Keio University, Tokyo, Japan

**Keywords:** asthma-COPD overlap, chronic obstructive pulmonary disease, eosinophil, IL-33, interferon-γ, multi-omics, statin, tumor necrosis factor-α

## Abstract

**Introduction:**

Elevated blood eosinophil levels in patients with chronic obstructive pulmonary disease (COPD) with or without asthma are linked to increased exacerbations and the effectiveness of inhaled corticosteroid treatment. This study aimed to delineate the inflammatory cellular properties of eosinophils in patients with asthma-COPD overlap (ACO) and eosinophilic COPD (eCOPD).

**Methods:**

Eosinophils were isolated from the peripheral blood of healthy volunteers, patients with non-eCOPD, and those with ACO/eCOPD. Multi-omics analysis involving transcriptomics, proteomics, and lipidomics was performed, followed by bioinformatic data analyses. In vitro experiments using eosinophils from healthy volunteers were conducted to investigate the molecular mechanisms underlying cellular alterations in eosinophils.

**Results:**

Proteomics and transcriptomics analyses revealed cellular characteristics in overall COPD patients represented by viral infection (elevated expression of sterol regulatory element-binding protein-1) and inflammatory responses (elevated levels of IL1 receptor-like 1, Fc epsilon receptor Ig, and transmembrane protein 176B). Cholesterol metabolism enzymes were identified as ACO/eCOPD-related factors. Gene Ontology and pathway enrichment analyses demonstrated the key roles of antiviral responses, cholesterol metabolism, and inflammatory molecules-related signaling pathways in ACO/eCOPD. Lipidomics showed the impaired synthesis of cyclooxygenase-derived mediators including prostaglandin E2 (PGE2) in ACO/eCOPD. In vitro assessment confirmed that IL-33 or TNF-α stimulation combined with IL-5 and IFN-γ stimulation induced cellular signatures in eosinophils in ACO/eCOPD. Atorvastatin, dexamethasone, and PGE2 differentially modulated these inflammatory changes.

**Discussion:**

ACO/eCOPD is associated with viral infection and an inflammatory milieu. Therapeutic strategies using statins and inhaled corticosteroids are recommended to control these pathogenic changes.

## 1 Introduction

Chronic obstructive pulmonary disease (COPD) is characterized by chronic airway inflammation and emphysema, typically marked by the accumulation of macrophages and neutrophils. The concurrent presence of airway eosinophilic inflammation in COPD has been previously reported on (1). In the general population, blood eosinophilia is positively associated with COPD and current smoking (2). Previous research has shown that 56% of patients with COPD have blood eosinophilia (≥150 cells/mL) (3). Patients with elevated blood eosinophil count in COPD experience more frequent exacerbations, and there exists a direct correlation between blood eosinophil counts and exacerbation frequency (4). Furthermore, lung tissue eosinophils increased in severe COPD (5). The therapeutic efficacy of inhaled corticosteroids is higher in patients with eosinophilic COPD (eCOPD) than that in those without eCOPD (non-eCOPD) (6–9). Despite these findings, the precise role of eosinophilic inflammation in COPD remains elusive. Therefore, the role of eosinophilic inflammation in COPD remains unclear.

Patients with COPD often have comorbid asthma. Patients with asthma-COPD overlap (ACO) present with frequent exacerbations and rapidly declining pulmonary function (10). Our previous study has established that blood eosinophil count, as opposed to neutrophil count, serves as a predictor of exacerbation (11). Thus, the quantitative differences and similarities in eosinophilic inflammation between COPD and asthma need to be clearly understood.

Studies on patients with allergic diseases have demonstrated inflammatory changes in eosinophils present in blood or tissues (12, 13). Type 2 cytokines, such as IL-5, play pivotal roles in these alterations. Our previous research has highlighted pro-inflammatory changes in eosinophils from patients with severe asthma and eosinophilic chronic sinusitis, accompanied by dysregulated fatty acid metabolism (14, 15). Activators for eosinophils in the local milieu include type 2 cytokines and microbial responses (15). At present, it is unclear whether eosinophils are activated, or which factors can activate these cells in COPD. Furthermore, the activation status of eosinophils and the factors driving their activation in COPD remain unclear.

In this study, we performed a comprehensive analysis of blood eosinophils from healthy volunteers and COPD patients with or without ACO and eCOPD using multi-omics analyses to elucidate the cellular characteristics of eosinophils in COPD.

## 2 Methods

### 2.1 Study population

Five healthy participants with no history of asthma or COPD (HP group), five patients without eCOPD (non-eCOPD group), and six patients with ACO and eCOPD (ACO/eCOPD group) were included in this study (**Supplementary Figure 1**). The characteristics of the participants are summarized in **Supplementary Table 1**. This study was approved by the Institutional Review Boards of RIKEN (Y2020-038) and Keio University School of Medicine (20200100). All participants provided written informed consent prior to participation. The study protocol is registered at UMIN clinical trials registry (UMIN000048174).

### 2.2 Isolation of eosinophils from human peripheral blood

Eosinophils were isolated from the peripheral blood of healthy volunteers and patients with COPD using the eosinophil isolation kit (Miltenyi Biotec, Bergisch Gladbach, Germany) following the manufacturer’s instructions. Briefly, a negative selection method using biotin-conjugated antibodies against various cell surface markers, including CD2, CD14, CD16, CD19, CD56, CD123, and CD235a, and microbeads conjugated to monoclonal anti-biotin antibodies was used.

### 2.3 RNA and protein extraction

Total RNAs and proteins were isolated from eosinophil lysates as described in the Materials and methods section of the online data supplement.

### 2.4 RNA sequencing analysis

RNA-seq libraries were prepared according to according to the instructions of the Quant Seq 3’ mRNA-seq library preparation kit FWD of Illumina (Lexogen, Vienna, Austria), sequenced, and analyzed as described in the Materials and methods section of the online data supplement.

### 2.5 Proteome analysis

The extracted proteins were digested into peptides and analyzed as described in the Materials and methods section of the online data supplement.

### 2.6 Targeted liquid chromatography-tandem mass spectrometry-based lipidomics

LC-MS/MS analysis was performed as described in a previous study (15) with technical modifications to the extraction protocol for fatty acid metabolites. Details are described in the Materials and methods section of the online data supplement.

### 2.7 *In vitro* treatment of peripheral blood eosinophils

Purified eosinophils (1.0 × 10^6^ cells/mL) were seeded in RPMI medium supplemented with 10% fetal bovine serum and 100 IU/mL of penicillin and cultured with 10 ng/mL type 2 cytokines (interleukin-4 (IL-4), IL-5, and IL-33), interferon (IFN)-α/γ, and tumor necrosis factor (TNF)-α (R&D Systems, Minneapolis, MN) for 3 or 24 h. Cells were pretreated with 100 μM atorvastatin (ATO), 10 nM dexamethasone (DEX), and 100 nM prostaglandin E_2_ (PGE_2_) for 15 min. Afterwards, the expression levels of mRNA (*FCER1G*, *MVD*, *SQLE*, *TMEM176B*, and *GGT5*) and cell surface antigens (FCER1G) were evaluated using quantitative reverse transcription (RT)-PCR and flow cytometric analyses. The details are described in the Materials and Methods section of the online data supplement.

### 2.8 Statistical analysis

Data are presented as mean ± standard error of the mean. Differences in lipid mediator biosynthesis, expression of protein and mRNA between the two groups were examined using repeated analysis of variance, followed by Dunnett’s or Tukey’s multiple comparison test. The expression of mRNA and surface antigens in eosinophils was also evaluated through repeated analysis of variance, followed by Dunnett’s or Tukey’s multiple comparisons tests. Data were analyzed using GraphPad Prism (version 9.0; GraphPad Software, San Diego, CA, USA). A *p*-value of < 0.05 was considered significant.

## 3 Results

### 3.1 Proteomic analysis of blood eosinophils from healthy participants and patients with COPD

**Figure 1:**
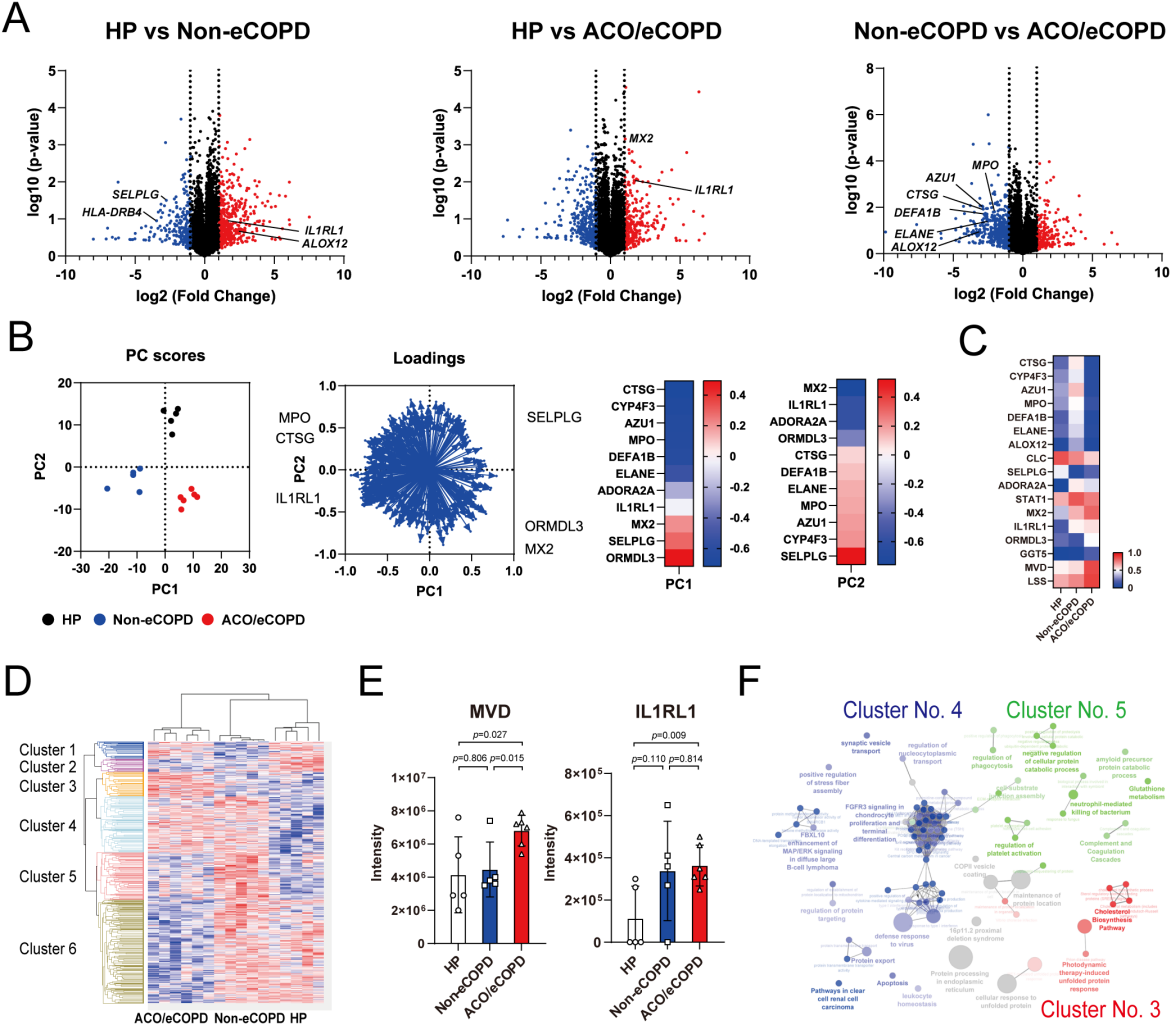
Proteomic analysis of peripheral blood eosinophils from healthy participants (HP) and patients with asthma-COPD overlap (ACO) and eosinophilic COPD (eCOPD) or without eCOPD (non-eCOPD). **(A)** Volcano plots of differentially expressed proteins among the three groups: HP vs non-eCOPD, HP vs ACO/eCOPD, and non-eCOPD vs ACO/eCOPD. The fold-change was plotted based on log_2_ fold change and log_10_ *P*-values. **(B)** Principal component analysis (PCA) of groups using representative proteins that distinguished them. Heatmap of PC1 and PC2 components of the proteins that clearly discriminated the differences among the groups in the PCA. **(C)** Heatmap showing the relative protein expression levels of all samples in the three groups from top to bottom. **(D)** Cluster classification of protein expression levels converted to Z-scores that presented statistically significant differences among the three groups and were classified into six clusters. These clusters include proteins with the highest expression levels in ACO/eCOPD (Cluster 3), ACO/eCOPD and non-eCOPD (Cluster 4), and non-eCOPD (Cluster 5) groups, respectively. **(E)** Protein expression levels of representative molecules classified into Clusters 3 and 5: molecules with ACO/eCOPD-specific upregulation and non-eCOPD-specific upregulation. **(F)** Gene Ontology enrichment analyses of genes in the three clusters (3, 4, and 5). Data show mean ± standard error of the mean; n = 5–6 for each group.

A total of 7388 proteins were identified in eosinophils, and the expression of 815 of these proteins differed significantly among HP, non-eCOPD, and ACO/eCOPD groups. A volcano plot using these data demonstrated different protein expression signatures among the three groups (**Figure 1A**). The representative proteins were selected and highlighted based on their relevance to eosinophil cell biology and the results of differential protein expression analysis between the groups. Subsequently, principal component analysis (PCA) using genes that showed differences in protein expression among the groups and identified the characteristic genes in different groups (**Figure 1B**). PC1 components indicated that anti-bacterial peptides, cathepsin G (CTSG), azurocidin 1 (AZU1), defensin alpha 1B (DEFA1B), neutrophil elastase (ELANE), and myeloperoxidase (MPO), were characteristic for non-eCOPD and ORMDL sphingolipid biosynthesis regulator 3 (ORMDL3), selectin P ligand (SELPLG), and MX Dynamin Like GTPase 2 (MX2), an anti-virus molecule, were specific for ACO/eCOPD group (**Figure 1B**). In addition, PC2 components showed a similar trend of anti-bacterial peptides and MPO and manifested a unique upregulation pattern of MX2 and interleukin 1 receptor-like 1 (IL1RL1), an IL-33 receptor, in both ACO/eCOPD and non-eCOPD groups (**Figure 1B**). A heat map was used to visualize the comparative expression levels of these molecules (**Figure 1C**). The characteristic changes were further demonstrated using cluster classification of the molecules, with statistically significant differences among the three groups (**Figure 1D**). Among the six clusters, Clusters 3 and 5 included the proteins with the highest expression levels in the ACO/eCOPD and non-eCOPD groups, respectively. These clusters included mevalonate diphosphate decarboxylase (MVD), lanosterol synthase (LSS), and IL1RL1 in Cluster 3; and signal transducer and activator of transcription 1 (STAT1) and MPO in Cluster 5 (**Figure 1E**, **Supplementary Figure 2**).

Enrichment analysis was performed to comprehensively evaluate these changes and understand the cellular characteristics of eosinophils in COPD. Wikipathway and KEGG pathway analyses indicated the importance of protein processing in the endoplasmic reticulum, cholesterol synthesis, and sterol regulatory element-binding protein (SREBP) signaling in Cluster 3 (**Supplementary Figure 3**). Upstream analysis of the proteins in Cluster 3 identified ribosomal protein S6 kinase A5 (RPS6KA5; MSK1) in STAT1 and Akt-JNK/ERK signaling pathways and mitogen-activated protein kinase kinase kinase 11 (MAP3K11; MLK3) in MAPK8/JNK, ERK, and NF-κB signaling pathways as upstream regulatory factors (**Supplementary Figure 4**).

Cluster classification by Gene Ontology (GO) enrichment analysis of each cluster revealed the characteristics of the three clusters— cholesterol synthesis in Cluster 3, antiviral activity in Cluster 4, and neutrophil-mediated bactericidal activity, platelet activation, and complement and coagulation factors in Cluster 5 (**Figure 1F**).

### 3.2 Transcriptomic analysis of blood eosinophils from healthy volunteers and patients with COPD

**Figure 2:**
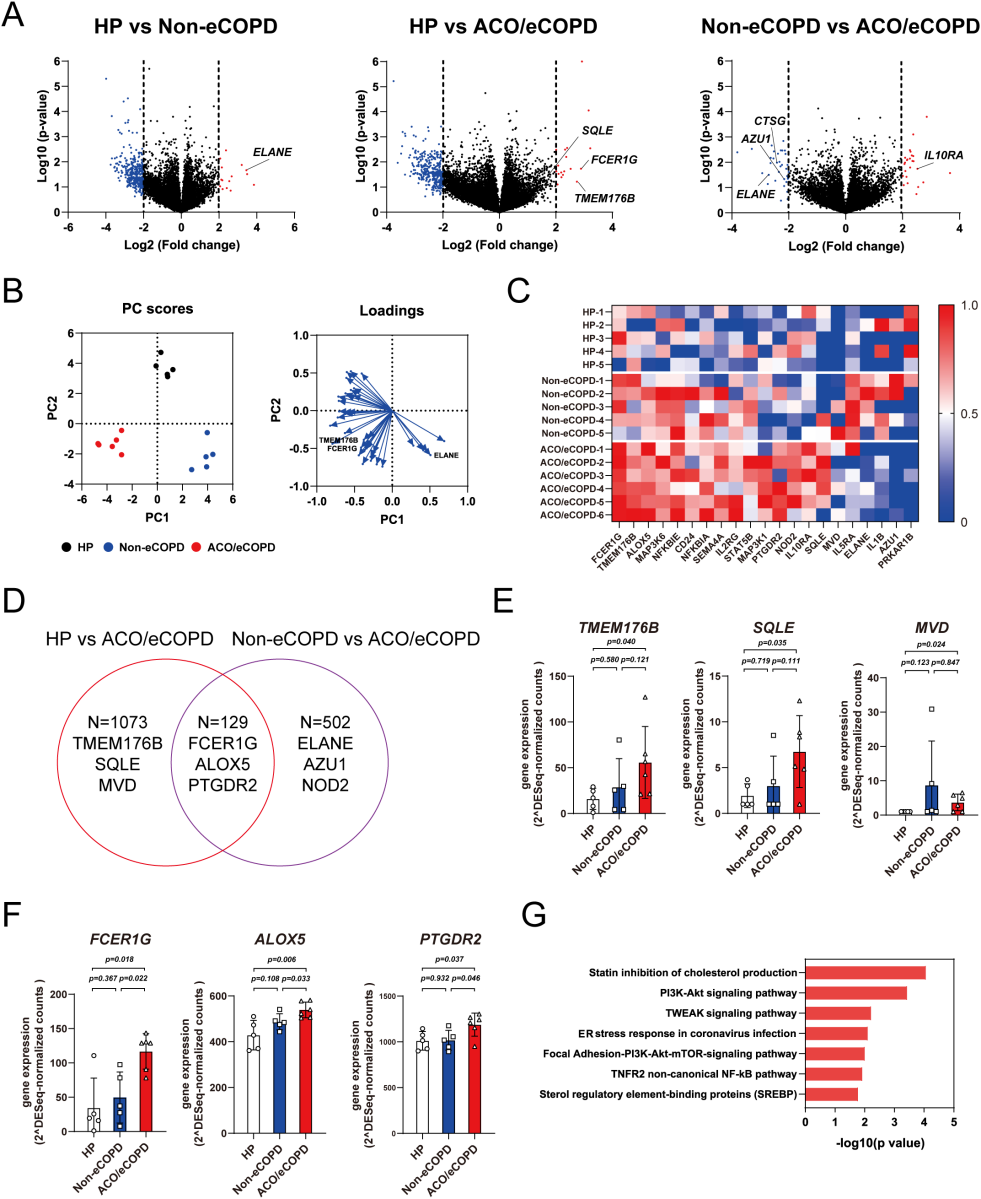
Transcriptomic analysis of peripheral blood eosinophils from healthy participants (HP) and patients with asthma-COPD overlap (ACO) and eosinophilic COPD (eCOPD) or without eosinophilic COPD (non-eCOPD). **(A)** Volcano plots of differentially expressed genes among the three groups; HP vs. non-eCOPD, HP vs. ACO/eCOPD, and non-eCOPD vs. ACO/eCOPD). Fold change was plotted based on log_2_ fold change and log_10_ *P*-values. **(B)** Principal component analysis (PCA) of groups using representative genes that distinguish them. Heatmap of PC1 and PC2 components of all genes in the PCA. **(C)** Heatmap showing representative gene expression levels of all samples in the three groups. **(D)** Venn diagram demonstrating differences in gene expression signatures among the three groups. **(E, F)** Gene expression levels of representative molecules classified into two groups: those with ACO/eCOPD-specific upregulation compared with HP **(E)** and both HP and non-eCOPD **(F)**. **(G)** Wiki pathway analysis annotated keyword classification and functional enrichment for upregulated molecules, specifically in ACO/eCOPD. Mean ± SEM, n = 5–6 for each group.

Next, RNA-seq was performed to evaluate the gene signatures of HP and patients with or without ACO and eCOPD, which identified 19788 protein-coding genes. A volcano plot demonstrated different gene expression signatures among the three groups (**Figure 2A**). The representative genes were selected and highlighted based on their relevance to eosinophil cell biology and the results of differential gene expression analysis between the groups. PCA using genes that showed significant differences in expression among the groups identified characteristics genes representing each group (**Figure 2B**). Among these, the expression of Fc epsilon receptor Ig (*FCER1G*), transmembrane protein 176 B (*TMEM176B*), and squalene epoxidase (*SQLE*) differed between the HP and eCOPD groups. Genes encoding antibacterial proteins *ELANE* and *AZU1* showed differential expression between the non-eCOPD and ACO/eCOPD groups (**Supplementary Figure 5**). As shown in **Figure 2C**, the heat map used to visualize the expression levels of representative genes also revealed the differences in their expression among the three groups. Among the protein-coding genes, 1202 and 632 genes showed higher expression in the ACO/eCOPD group than those in the HP and non-eCOPD groups, respectively (**Figure 2D**). The genes showing differential expression in HP vs ACO/eCOPD groups included *TMEM176B, SQLE, MVD, FCER1G, ALOX5*, and prostaglandin D2 receptor 2 (*PTGDR2*) (**Figures 2E, F**). Of these, the expression levels of 129 genes, including *FCER1G, ALOX5*, and *PTGDR2*, were higher in patients with ACO and eCOPD than those in both healthy volunteers and patients without ACO and eCOPD (**Figure 2F**). To elucidate the correlation between the proteomic and transcriptomic data of human eosinophils, an integrated data analysis was conducted. As shown in **Supplementary Table 2** and **Supplementary Figure 6**, the protein and gene expressions of representative molecules, including FCER1G, exhibited statistically significant correlations; however, not all molecules demonstrated such correlations.

To identify the functional relevance of differentially expressed genes, pathway analysis of the genes showing significantly elevated expression in patients with or without ACO/eCOPD was performed to identify specific signaling pathways in non-eCOPD and ACO/eCOPD groups. This analysis identified that those in both non-eCOPD and ACO/eCOPD groups were related to TNF-related factors, MAPK, oxidative damage, interferon, and PI3K-Akt (**Supplementary Figure 7**) and those only in ACO/eCOPD group were linked to cholesterol metabolism, PI3K-Akt-mTOR, TNF downstream signaling, endoplasmic reticulum stress, and nuclear factor-kappa B (NF-κB) (**Figure 2G**).

These results confirmed that IL-33, TNF, NF-κB signaling, and the IFN-STAT1 pathway with antiviral activity and cholesterol metabolism are uniquely related to ACO and eCOPD. Additionally, these findings also suggest that *FCER1G, TMEM176B, SQLE*, and *MVD* are the key genes for characterizing the functional changes in ACO/eCOPD group.

### 3.3 Lipidomic analysis of blood eosinophils from healthy participants and patients with COPD

**Figure 3:**
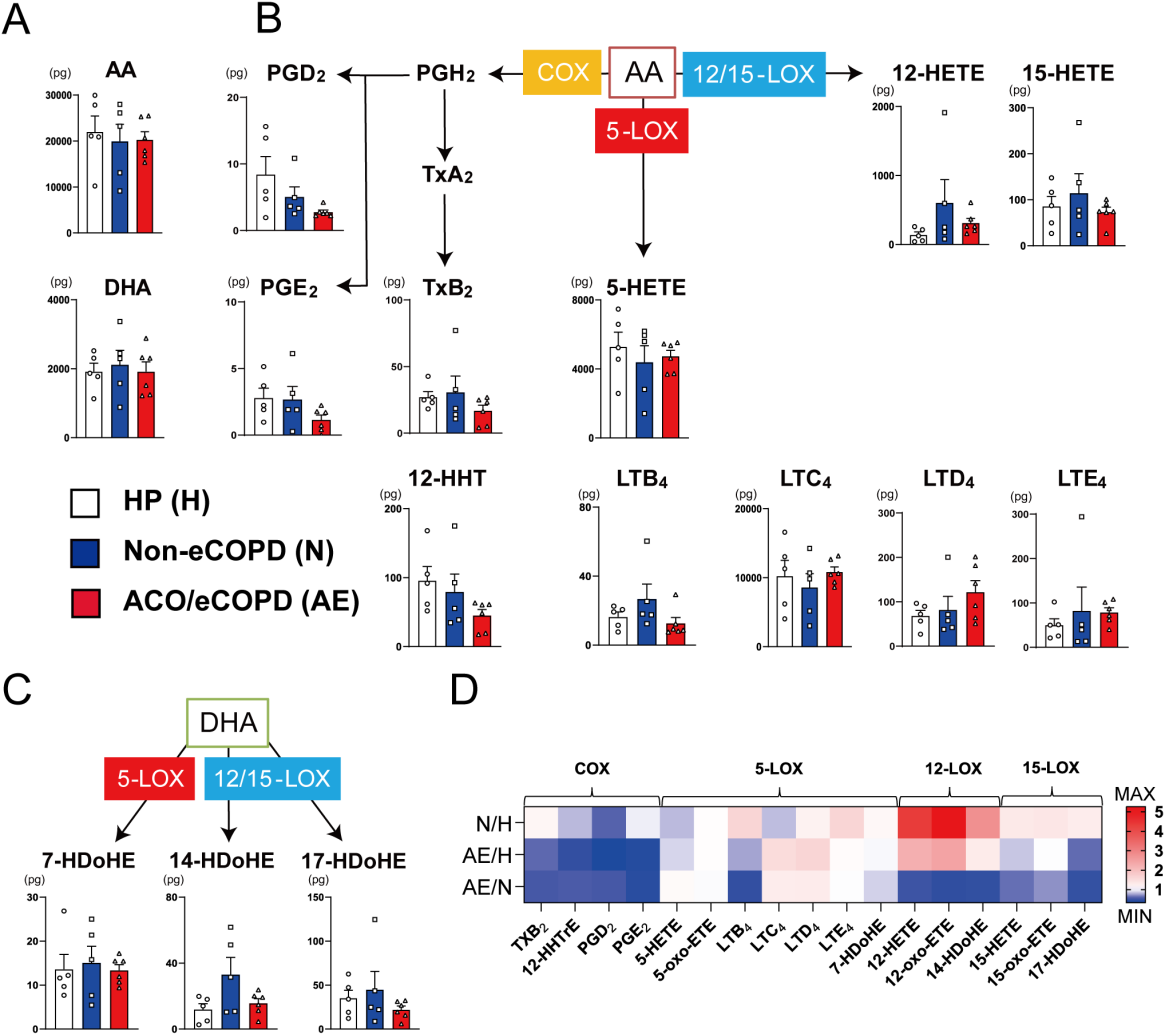
Lipidomic analysis of peripheral blood eosinophils from healthy participants (HP) and patients with asthma-COPD overlap (ACO) and eosinophilic COPD (eCOPD) or without eosinophilic COPD (non-eCOPD). **(A)** Lipidomic analysis of arachidonic acid (AA) and docosahexaenoic Acid (DHA). **(B)** Lipidomic analysis of 5-lipoxygenase (5-LOX) (leukotrienes (LTs) and 5-hydroxy eicosatetraenoic acid (5-HETE)), 12/15-lipoxygenase (12/15-LOX) (12-HETE and 15-HETE), and cyclooxygenase (COX) (prostaglandins (PGs) and thromboxanes (Txs))-mediated mediators derived from AA. **(C)** Lipidomic analysis of 5-LOX (7-hydroxy docosahexaenoic acid (7-HDoHE))-and 12/15-LOX (14-HDoHE and 17-HDoHE)-mediated mediators derived from DHA. **(D)** Heatmap representing the comparative evaluation of lipidomic profiles among the three groups (upper row, non-eCOPD: HP; middle row, ACO/eCOPD: HP; and lower row, ACO/eCOPD:non-eCOPD). Mean ± SEM, n = 5–6 for each group.

Lipidomic analysis was performed to assess fatty acid metabolism in eosinophils of patients with COPD. The release of fatty acids, including arachidonic acid and docosahexaenoic acid, remained unchanged in all groups (**Figure 3A**). In contrast, fatty acid-related enzyme-regulating synthesis patterns differed among the groups (**Figures 3B, C**). The synthesis of cyclooxygenase (COX)-related mediators, including PGE_2_, PGD_2_, and thromboxane, was also decreased in the ACO/eCOPD group. The synthesis of leukotriene D_4_ (LTD_4_) showed an increasing trend, while those of 15-lipoxygenase (LOX)-derived metabolites (15-hydroxyeicosatetraenoic acid (15-HETE) and 17- hydroxydocosahexaenoic acid (17-HDoHE)) were decreased in the ACO/eCOPD group. In contrast, 12-LOX-related metabolites (12-HETE and 14-HDoHE) and LTB_4_ showed increasing trends in the non-eCOPD group. Furthermore, the heat map showing the ratios of released metabolites to compare every group showed the selectively defective synthesis of PGE_2_ in the ACO/eCOPD group (**Figure 3D**).

### 3.4 *In vitro* experiments using blood eosinophils from healthy volunteers

**Figure 4:**
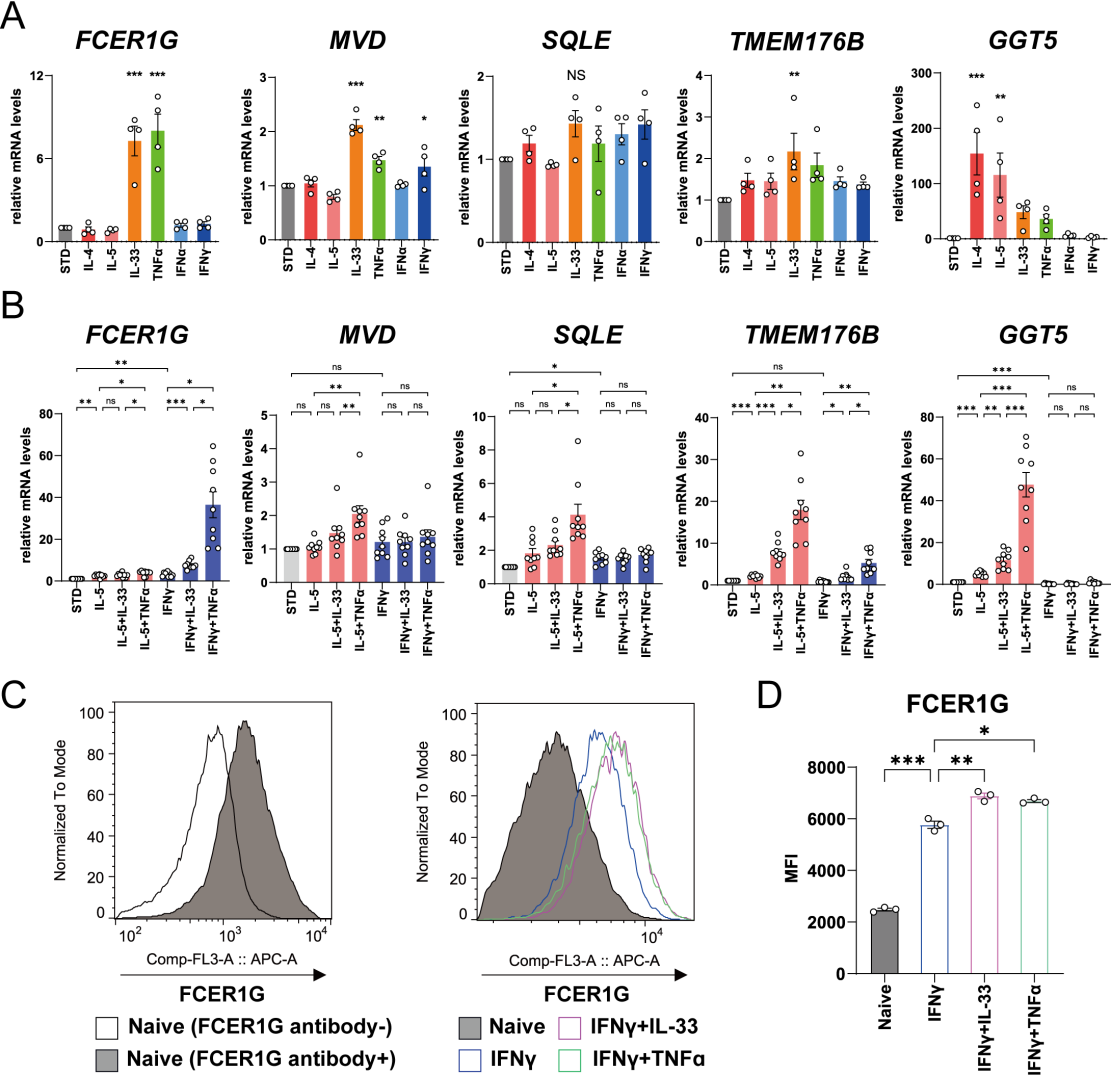
Regulatory mechanism of mRNA and protein expressions of the molecules in human eosinophils upregulated in patients with asthma-COPD overlap (ACO) and eosinophilic COPD (eCOPD). **(A, B)** Comparative analysis of the mRNA expression of the genes (*FCER1G, MVD, SQLE, TMEM176B*, and *GGT5*) using quantitative RT-PCR of the cells stimulated with 10 ng/mL of various cytokines (IL-4, IL-5, IL-33, TNF-α , IFN-α, and IFN-γ) for 3 h **(A)** or 24 h **(B)**. **(C)** Histogram of protein expression of FCER1G on the cell surface of the cells unstimulated or stimulated with IFN-γ combined with IL-33/TNF-α for 72 h. **(D)** Mean fluorescence intensity (MFI) of protein expression of FCER1G on the cell surface of human eosinophils unstimulated or stimulated with IFN-γ combined with IL-33/TNF-α for 72 h. Mean ± SEM, n = 3–9 for each group. *P < 0.05, **P < 0.01, ***P < 0.001. Data are representative of at least three independent experiments.

Next, we performed an *in vitro* experiment to confirm the results of multi-omics analyses. Of the genes upregulated in patients with ACO/eCOPD, four genes (*FCER1G*, a high-affinity IgE receptor; *TMEM176B*, a transmembrane protein; *SQLE* and *MVD*, encoding the enzymes for cholesterol metabolism), in addition to gamma-glutamyl transferase 5 (*GGT5*), which is typically upregulated by stimulation with type 2 cytokines (15) were selected to evaluate their regulatory mechanisms. Short-term stimulation with cytokines for 3 h significantly upregulated the mRNA expression of these genes (**Figure 4A**, **Supplementary Figure 8**). IL-33 upregulated *FCER1G*, *MVD*, and *TMEM176B* expression, whereas TNF-α upregulated the expression of *FCER1G* and *MVD*. IFN-γ upregulated the expression of *MVD*. However, stimulation with IL-4- and IL-5 upregulated *GGT5*, while none of the cytokines altered the expression of *SQLE* in the present study.

Long-term stimulation of eosinophils with IL-33 or TNF-α in combination with IL-5 or IFN-γ for 24 h demonstrated that IL-5 elevated the expression of *TMEM176B* and *GGT5* mRNAs, IFN-γ upregulated *FCER1G* and *SQLE* mRNA, and TNF-α and IL-33 synergistically upregulated the mRNA expression of these genes (**Figure 4B**). *FCER1G* expression was specifically elevated by stimulation with IFN-γ and TNF-α. Flowcytometric analysis demonstrated cell surface expression of *FCER1G* on blood eosinophils of healthy volunteers and its upregulation by combined stimulation with IFN-γ and IL-33 or TNF-α (**Figures 4C, D**, **Supplementary Figure 9**). These findings suggest that IL-33, TNF-α, and IFN-γ, in addition to IL-5, are the key cytokines for the activation of eosinophils in eCOPD.

### 3.5 Effects of statin, DEX, and PGE2 on eosinophil activation

To investigate whether pharmacological interventions and physiological factors suppress the cellular activation of eosinophils in patients with ACO and eCOPD, we examined the effects of statins, corticosteroids, and PGE_2_ on these activities based on the findings of multi-omics analysis and the usefulness of corticosteroids in COPD (6, 7).

**Figure 5:**
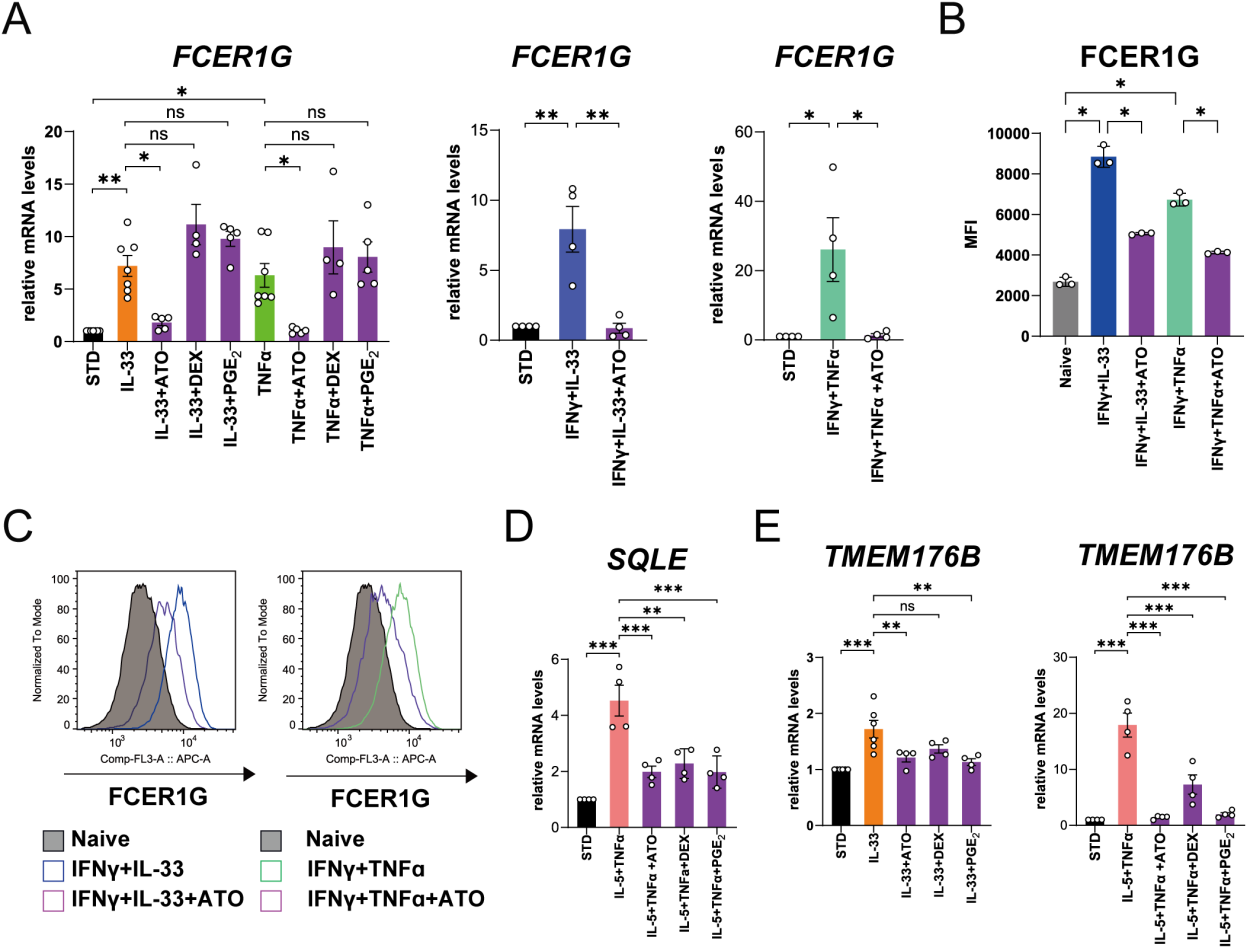
Effects of atorvastatin (ATO), dexamethasone (DEX), and prostaglandin E_2_ (PGE_2_) on mRNA and protein expressions of the representative molecules in human eosinophils upregulated in patients with asthma-COPD overlap (ACO) and eosinophilic COPD (eCOPD). **(A)** The effects of ATO, DEX, and PGE_2_ on *FCER1G* mRNA expression in the cells stimulated with IL-33 or TNF-α for 3 h or IFN-γ plus IL-33 or IFN-γ plus TNF-α for 24 h. **(B)** Mean fluorescence intensity (MFI) of protein expressions of FCER1G on cell surface of human eosinophils stimulated with IFN-γ combined with IL-33/TNF-α pretreated with/without ATO for 72 h. **(C)** Histogram of protein expression of FCER1G on cell surface of the cells stimulated with IFN-γ plus IL-33 or IFN-γ plus TNF-α pretreated with/without ATO for 72 h. **(D)** The effects of ATO, DEX, and PGE_2_ on *SQLE* mRNA expression in the cells stimulated with IL-5 plus TNF-α for 24 h. **(E)** The effects of ATO, DEX, and PGE_2_ on *TMEM176B* mRNA expression in the cells stimulated with IL-33 for 3 h or IL-5 plus TNF-α for 24 h. Mean ± SEM, n = 4–7 for each group. *P < 0.05, **P < 0.01, ***P < 0.001. Data are representative of at least three independent experiments.

ATO, DEX, and PGE_2_ inhibited the IL-4- or IL-5-induced upregulation of *GGT5* mRNA expression at concentrations of 100 μM, 10 nM, and 100 nM, respectively (**Supplementary Figures 10** and **11**). In contrast, IL-33 and TNF-α-induced upregulation of *FCER1G* mRNA expression was selectively suppressed by ATO (**Figure 5A**). ATO also inhibited *FCER1G* upregulation by combined stimulation with IFN-γ and IL-33 or TNF-α (**Figure 5A**). Furthermore, flow cytometry confirmed the inhibitory effects of ATO on the expression of cell surface proteins (**Figures 5B, C**). Upregulation of *SQLE* and *TMEM176B* induced by IL-5 plus TNF-α or IL-33 stimulation was suppressed by ATO, DEX, and PGE_2_ (**Figures 5D, E**).

Collectively, these results indicate that statins and steroids have differential therapeutic effects and that PGE_2_ possesses physiological anti-inflammatory effects on cellular activation in ACO and eCOPD.

## 4 Discussion

This study presents a comprehensive evaluation revealing altered cellular properties of eosinophils in patients with COPD, especially ACO and eCOPD. The bioinformatics approach underscores the involvement of the antiviral response and cholesterol metabolism, particularly in ACO and eCOPD. Moreover, inflammatory cytokines IL-33, TNF-α, and IFN-γ potentially activate eosinophils in overall COPD, signaling a distinct regulatory mechanism compared to IL-5. Dysregulation of COX metabolism, particularly in ACO/eCOPD group, and the self-regulatory function of its downstream mediator, PGE_2_, were identified. *In vitro* screening indicated the suppressive effects of statins, in addition to steroids, on observed inflammatory changes in ACO and eCOPD.

Viral and bacterial infections are the major causes of COPD exacerbation. However, sputum eosinophils are only increased by viral infection during exacerbations (16), suggesting a close link between viral infection and eosinophilic inflammation in COPD. Viruses enter airway epithelial and immune cells, including alveolar macrophages, which induce innate immune responses via IFN production. Sufficient amounts of IL-33 are released from airway epithelial cells during viral infection (17). The findings suggest that IFN-γ- and IL-33-induced inflammatory responses during viral infection may induce eosinophil activation in overall patients with COPD, particularly during exacerbations.

IFNs activate eosinophils with pro-survival effects. Among the IFNs, IFN-γ, can augment cytokine production and modulate adhesion molecule expressions (18). A previous study on asthma reported that IFNs promote cysteinyl LT production by eosinophils (19). In COPD, IFN-γ has been detected as a major pro-inflammatory cytokine whose expression correlates with disease severity (20–22) and is commonly related to both COPD and asthma (23). In addition, our recent study demonstrated that IFN-γ activates human eosinophils, enhancing their responsiveness to IL-5 (24). These findings suggest that IFN-γ may serve as a key activator of eosinophils in the broader COPD population, underscoring the importance of preventive strategies against viral infections.

TNF-α is a pro-inflammatory cytokine produced mainly by macrophages and plays a pivotal role in the pathogenesis of COPD. Its production correlates with disease severity and increases during exacerbations (21, 25). TNF-α can activate eosinophils to induce degranulation and reactive oxygen species generation (26). Additionally, TNF-α and IFN-γ have synergistic effects on cellular activation of eosinophils (27). This study showed that eosinophils in patients with COPD could be activated by TNF-α, suggesting its pro-inflammatory roles in eosinophil activation in COPD in addition to IFN-γ, contributing to the pathogenesis of COPD comorbidities such as osteoporosis and cardiovascular disease.

IL-33 is a nuclear-localized cytokine mainly found in airway epithelial cells. Exposure to allergens and microorganisms induces their release from the cells. IL-33 mainly activates type 2 innate lymphoid cells (ILC2) that release large amounts of type 2 cytokines and eosinophils (28, 29). Its expression increases with the severity of asthma and COPD (30, 31). Of note, IL-13 levels with lower respiratory function were positively correlated with IL-33 expression in COPD (17, 32). In addition, our previous study also demonstrated that IL-13 increased IL1RL1 expression on human eosinophils, potentially augmenting IL-13/IL-33 axis-dependent inflammation (33). In animal experiments, IL-33 released by viral infections or cigarette smoking activated NK cells and macrophages, not ILC2, to induce IFN production, which contributed to emphysema development (17). In the present study, eosinophils in patients with ACO and eCOPD had increased expression of IL1RL1 and IL-33-induced upregulation of gene expression characteristics in ACO and eCOPD, suggesting the importance of IL-33 in the pathogenesis of ACO and eCOPD. Biologics targeting IFN-γ, TNF-α, and IL-33 are not yet available for asthma and COPD patients. However, dupilumab, a monoclonal antibody against IL-4 receptor alpha, is used as a therapeutic option for severe asthma and has demonstrated efficacy in patients with eCOPD (34, 35). Based on our previous study (33), dupilumab may be a viable option for managing eCOPD by controlling the IL-13/IL-33 axis.

IL-5 is a potent pro-survival and eosinophil-activating factor. IL-5-targeting biologics are effective therapeutic agents for reducing eosinophilia (36). Clinical trials using these agents for patients with COPD have shown that their therapeutic efficacies are inconclusive, although mepolizumab was effective in a minority of patients with COPD with blood eosinophilia (37, 38). Our study indicated that IL-5 specifically upregulates the expression of cholesterol metabolism-related enzymes (MVD and SQLE) in eosinophils of patients with ACO and eCOPD. Identifying patients with ACO and/or eCOPD in whom IL-5 is involved in the pathogenesis may optimize therapeutic strategies using anti-IL-5 and IL-5 receptor antibodies.

Dyslipidemia is a common comorbidity in patients with COPD. Statins are the first-line treatment for hyperlipidemia. Interestingly, previous studies have reported that statins reduce the risk of exacerbations and death in COPD (39, 40). However, this is not necessarily effective in all patients (41). In our study, cholesterol metabolism was enhanced only in patients with ACO/eCOPD group, and atorvastatin showed an anti-inflammatory effect on the changes in eosinophils. Therefore, therapeutic strategies targeting cholesterol metabolism in eosinophils using statins may be effective, particularly in patients with ACO and eCOPD, who frequently have dyslipidemia as a comorbidity.

COX metabolism, with higher levels of PGE_2_, is enhanced in patients with COPD, especially during exacerbations (42). *In vitro* experiments have demonstrated that PGE_2_-EP4-mediated signaling showed anti-inflammatory effects on eosinophils (43) and elicits bronchodilation (44). Lipidomic analysis indicated that COX metabolism was impaired in ACO/eCOPD group at the cellular level. Further investigations are required to elucidate the underlying mechanisms and therapeutic potential and risks of targeting the COX pathway in this metabolic cascade.

In this study, multi-omics analysis identified FCER1G as a key molecule with correlation between its protein and gene expression levels, that distinguishes ACO/eCOPD patients from healthy participants and non-eCOPD patients. Investigating the regulatory mechanisms of FCER1G in eosinophils contributed to understanding the inflammatory milieu and therapeutic potential of specific molecules. While there may be weak or no correlation between protein and gene expression levels of specific molecules in granulocytes, including neutrophils and eosinophils, due to variability and the nature of granule and membranous proteins (15, 45), this methodology can detect broad immune cell abnormalities and reveal disease pathophysiology in inflammatory diseases. Proteomic analysis has been demonstrated in many studies to have high quantification ability, sensitivity, and reproducibility (46–50). However, further research using a larger patient population is required to confirm the results of this study.

In summary, the multi-omics analysis revealed cellular changes in blood eosinophils in patients with COPD, especially ACO and eCOPD. These changes are induced mainly by IL-33, TNF-α, and IFN-γ in addition to IL-5. Viral infections may be important to trigger cellular activation via these cytokines. Statins could be a potential treatment option for ACO and eCOPD, in addition to inhaled corticosteroids. Further clinical research is required to develop and optimize therapeutic strategies that target eosinophilic inflammation in patients with ACO and eCOPD.

## Data Availability

Proteomic data used in this study has been deposited to the ProteomeXchange Consortium via the jPOST partner repository (https://www.proteomexchange.org/) with the dataset identifier PXD052550. The RNA-seq data generated in this study are available in the DDBJ (DNA Data Bank of Japan: https://www.ddbj.nig.ac.jp/index-e.html) database under BioProject PRJDB18141 with GEA (Genomic Expression Archive) E-GEAD-802. MS data in lipidomic analysis are available at the DROP Met section of the RIKEN PRIMe (https://prime.psc.riken.jp/menta.cgi/prime/drop_index) via the index DM0059. Further inquiries can be directed to the corresponding author.

## References

[1] McDonald VM Higgins I Wood LG Gibson PG. Multidimensional assessment and tailored interventions for COPD: respiratory utopia or common sense? Thorax. (2013) 68:691–4. doi: 10.1136/thoraxjnl-2012-202646 PMC3711365 23503624

[2] Hartl S Breyer MK Burghuber OC Ofenheimer A Schrott A Urban MH. Blood eosinophil count in the general population: typical values and potential confounders. Eur Respir J. (2020) 55. doi: 10.1183/13993003.01874-2019 32060069

[3] Barnes N Ishii T Hizawa N Midwinter D James M Hilton E. The distribution of blood eosinophil levels in a Japanese COPD clinical trial database and in the rest of the world. Int J Chron Obstruct Pulmon Dis. (2018) 13:433–40. doi: 10.2147/COPD.S144108 PMC5799851 29440882

[4] Yun JH Lamb A Chase R Singh D Parker MM Saferali A. Blood eosinophil count thresholds and exacerbations in patients with chronic obstructive pulmonary disease. J Allergy Clin Immunol. (2018) 141:2037–47.e10. doi: 10.1016/j.jaci.2018.04.010 29709670 PMC5994197

[5] Jogdand P Siddhuraj P Mori M Sanden C Jönsson J Walls AF. Eosinophils, basophils and type 2 immune microenvironments in COPD-affected lung tissue. Eur Respir J. (2020) 55. doi: 10.1183/13993003.00110-2019 PMC7236868 32060064

[6] Pascoe S Barnes N Brusselle G Compton C Criner GJ Dransfield MT. Blood eosinophils and treatment response with triple and dual combination therapy in chronic obstructive pulmonary disease: analysis of the IMPACT trial. Lancet Respir Med. (2019) 7:745–56. doi: 10.1016/S2213-2600(19)30190-0 31281061

[7] Bafadhel M Peterson S De Blas MA Calverley PM Rennard SI Richter K. Predictors of exacerbation risk and response to budesonide in patients with chronic obstructive pulmonary disease: a *post-hoc* analysis of three randomised trials. Lancet Respir Med. (2018) 6:117–26. doi: 10.1016/S2213-2600(18)30006-7 29331313

[8] Suissa S Dell'Aniello S Ernst P. Comparative effectiveness of LABA-ICS versus LAMA as initial treatment in COPD targeted by blood eosinophils: a population-based cohort study. Lancet Respir Med. (2018) 6:855–62. doi: 10.1016/S2213-2600(18)30368-0 30343028

[9] Singh D Bafadhel M Brightling CE Sciurba FC Curtis JL Martinez FJ. Blood eosinophil counts in clinical trials for chronic obstructive pulmonary disease. Am J Respir Crit Care Med. (2020) 202:660–71. doi: 10.1164/rccm.201912-2384PP PMC7462391 32186896

[10] Sin DD Miravitlles M Mannino DM Soriano JB Price D Celli BR. What is asthma-COPD overlap syndrome? Towards a consensus definition from a round table discussion. Eur Respir J. (2016) 48:664–73. doi: 10.1183/13993003.00436-2016 27338195

[11] Mochimaru T Chubachi S Irie H Sakurai K Okuzumi S Sunata K. Blood eosinophil and neutrophil counts differentially identify frequent exacerbation in patients with COPD with physician-diagnosed asthma and COPD. Allergol Int. (2021) 70:255–7. doi: 10.1016/j.alit.2020.09.004 33087300

[12] Johansson MW. Eosinophil activation status in separate compartments and association with asthma. Front Med (Lausanne). (2017) 4:75. doi: 10.3389/fmed.2017.00075 28660189 PMC5466952

[13] Miyata J Fukunaga K Kawashima Y Ohara O Arita M. Cysteinyl leukotriene metabolism of human eosinophils in allergic disease. Allergol Int. (2020) 69:28–34. doi: 10.1016/j.alit.2019.06.002 31248811

[14] Miyata J Fukunaga K Iwamoto R Isobe Y Niimi K Takamiya R. Dysregulated synthesis of protectin D1 in eosinophils from patients with severe asthma. J Allergy Clin Immunol. (2013) 131:353–60.e1-2. doi: 10.1016/j.jaci.2012.07.048 23006546

[15] Miyata J Fukunaga K Kawashima Y Watanabe T Saitoh A Hirosaki T. Dysregulated fatty acid metabolism in nasal polyp-derived eosinophils from patients with chronic rhinosinusitis. Allergy. (2019) 74:1113–24. doi: 10.1111/all.2019.74.issue-6 30667533

[16] Papi A Bellettato CM Braccioni F Romagnoli M Casolari P Caramori G. Infections and airway inflammation in chronic obstructive pulmonary disease severe exacerbations. Am J Respir Crit Care Med. (2006) 173:1114–21. doi: 10.1164/rccm.200506-859OC 16484677

[17] Kearley J Silver JS Sanden C Liu Z Berlin AA White N. Cigarette smoke silences innate lymphoid cell function and facilitates an exacerbated type I interleukin-33-dependent response to infection. Immunity. (2015) 42:566–79. doi: 10.1016/j.immuni.2015.02.011 25786179

[18] Dajotoy T Andersson P Bjartell A Löfdahl CG Tapper H Egesten A. Human eosinophils produce the T cell-attracting chemokines MIG and IP-10 upon stimulation with IFN-gamma. J Leukoc Biol. (2004) 76:685–91. doi: 10.1189/jlb.0803379 15197236

[19] Steinke JW Liu L Huyett P Negri J Payne SC Borish L. Prominent role of IFN-γ in patients with aspirin-exacerbated respiratory disease. J Allergy Clin Immunol. (2013) 132:856–65.e1-3. doi: 10.1016/j.jaci.2013.05.008 23806637 PMC3788084

[20] Kang MJ Lee CG Lee JY Dela Cruz CS Chen ZJ Enelow R. Cigarette smoke selectively enhances viral PAMP- and virus-induced pulmonary innate immune and remodeling responses in mice. J Clin Invest. (2008) 118:2771–84. doi: 10.1172/JCI32709 PMC2483678 18654661

[21] Paats MS Bergen IM Hoogsteden HC van der Eerden MM Hendriks RW. Systemic CD4+ and CD8+ T-cell cytokine profiles correlate with GOLD stage in stable COPD. Eur Respir J. (2012) 40:330–7. doi: 10.1183/09031936.00079611 22183488

[22] Silver JS Kearley J Copenhaver AM Sanden C Mori M Yu L. Inflammatory triggers associated with exacerbations of COPD orchestrate plasticity of group 2 innate lymphoid cells in the lungs. Nat Immunol. (2016) 17:626–35. doi: 10.1038/ni.3443 PMC5345745 27111143

[23] Ghebre MA Pang PH Diver S Desai D Bafadhel M Haldar K. Biological exacerbation clusters demonstrate asthma and chronic obstructive pulmonary disease overlap with distinct mediator and microbiome profiles. J Allergy Clin Immunol. (2018) 141:2027–36.e12. doi: 10.1016/j.jaci.2018.04.013 29709671 PMC5986707

[24] Sasaki H Miyata J Kawashima Y Konno R Ishikawa M Hasegawa Y. Distinct roles of types 1 and 2 interferons in human eosinophil regulation: A multi-omics analysis. Allergy. (2024). doi: 10.1111/all.16215 38958441

[25] Aaron SD Angel JB Lunau M Wright K Fex C Le Saux N. Granulocyte inflammatory markers and airway infection during acute exacerbation of chronic obstructive pulmonary disease. Am J Respir Crit Care Med. (2001) 163:349–55. doi: 10.1164/ajrccm.163.2.2003122 11179105

[26] Slungaard A Vercellotti GM Walker G Nelson RD Jacob HS. Tumor necrosis factor alpha/cachectin stimulates eosinophil oxidant production and toxicity towards human endothelium. J Exp Med. (1990) 171:2025–41. doi: 10.1084/jem.171.6.2025 PMC2187940 1972179

[27] Williamson BD Carswell EA Rubin BY Prendergast JS Old LJ. Human tumor necrosis factor produced by human B-cell lines: synergistic cytotoxic interaction with human interferon. Proc Natl Acad Sci U S A. (1983) 80:5397–401. doi: 10.1073/pnas.80.17.5397 PMC384263 6193516

[28] Kabata H Moro K Koyasu S. The group 2 innate lymphoid cell (ILC2) regulatory network and its underlying mechanisms. Immunol Rev. (2018) 286:37–52. doi: 10.1111/imr.2018.286.issue-1 30294963

[29] Cherry WB Yoon J Bartemes KR Iijima K Kita H. A novel IL-1 family cytokine, IL-33, potently activates human eosinophils. J Allergy Clin Immunol. (2008) 121:1484–90. doi: 10.1016/j.jaci.2008.04.005 PMC2821937 18539196

[30] Gaurav R Poole JA. Interleukin (IL)-33 immunobiology in asthma and airway inflammatory diseases. J Asthma. (2022) 59:2530–8. doi: 10.1080/02770903.2021.2020815 PMC9234100 34928757

[31] Joo H Park SJ Min KH Rhee CK. Association between plasma interleukin-33 level and acute exacerbation of chronic obstructive pulmonary disease. BMC Pulm Med. (2021) 21:86. doi: 10.1186/s12890-021-01423-8 33722239 PMC7962403

[32] Byers DE Alexander-Brett J Patel AC Agapov E Dang-Vu G Jin X. Long-term IL-33-producing epithelial progenitor cells in chronic obstructive lung disease. J Clin Invest. (2013) 123:3967–82. doi: 10.1172/JCI65570 PMC3754239 23945235

[33] Sunata K Miyata J Kawashima Y Konno R Ishikawa M Hasegawa Y. Multiomics analysis identified IL-4-induced IL1RL1^high^ eosinophils characterized by prominent cysteinyl leukotriene metabolism. J Allergy Clin Immunol. (2024). doi: 10.1016/j.jaci.2024.07.012 39067484

[34] Bhatt SP Rabe KF Hanania NA Vogelmeier CF Bafadhel M Christenson SA. Dupilumab for COPD with blood eosinophil evidence of type 2 inflammation. N Engl J Med. (2024) 390:2274–83. doi: 10.1056/NEJMoa2401304 38767614

[35] Bhatt SP Rabe KF Hanania NA Vogelmeier CF Cole J Bafadhel M. Dupilumab for COPD with type 2 inflammation indicated by eosinophil counts. N Engl J Med. (2023) 389:205–14. doi: 10.1056/NEJMoa2303951 37272521

[36] Brusselle GG Koppelman GH. Biologic therapies for severe asthma. N Engl J Med. (2022) 386:157–71. doi: 10.1056/NEJMra2032506 35020986

[37] Criner GJ Celli BR Brightling CE Agusti A Papi A Singh D. Benralizumab for the prevention of COPD exacerbations. N Engl J Med. (2019) 381:1023–34. doi: 10.1056/NEJMoa1905248 31112385

[38] Pavord ID Chanez P Criner GJ Kerstjens HAM Korn S Lugogo N. Mepolizumab for eosinophilic chronic obstructive pulmonary disease. N Engl J Med. (2017) 377:1613–29. doi: 10.1056/NEJMoa1708208 28893134

[39] Søyseth V Brekke PH Smith P Omland T. Statin use is associated with reduced mortality in COPD. Eur Respir J. (2007) 29:279–83. doi: 10.1183/09031936.00106406 17050558

[40] Schenk P Spiel AO Hüttinger F Gmeiner M Fugger J Pichler M. Can simvastatin reduce COPD exacerbations? A randomised double-blind controlled study. Eur Respir J. (2021) 58. doi: 10.1183/13993003.01798-2020 33574076

[41] Criner GJ Connett JE Aaron SD Albert RK Bailey WC Casaburi R. Simvastatin for the prevention of exacerbations in moderate-to-severe COPD. N Engl J Med. (2014) 370:2201–10. doi: 10.1056/NEJMoa1403086 PMC4375247 24836125

[42] Drozdovszky O Barta I Antus B. Sputum eicosanoid profiling in exacerbations of chronic obstructive pulmonary disease. Respiration. (2014) 87:408–15. doi: 10.1159/000358099 24714447

[43] Mita H Hasegawa M Higashi N Akiyama K. Characterization of PGE2 receptor subtypes in human eosinophils. J Allergy Clin Immunol. (2002) 110:457–9. doi: 10.1067/mai.2002.127001 12209094

[44] Buckley J Birrell MA Maher SA Nials AT Clarke DL Belvisi MG. EP4 receptor as a new target for bronchodilator therapy. Thorax. (2011) 66:1029–35. doi: 10.1136/thx.2010.158568 PMC3221321 21606476

[45] Kawashima Y Miyata J Watanabe T Shioya J Arita M Ohara O. Proteogenomic analyses of cellular lysates using a phenol-guanidinium thiocyanate reagent. J Proteome Res. (2019) 18:301–8. doi: 10.1021/acs.jproteome.8b00609 30394753

[46] Aebersold R Burlingame AL Bradshaw RA. Western blots versus selected reaction monitoring assays: time to turn the tables? Mol Cell Proteomics. (2013) 12:2381–2. doi: 10.1074/mcp.E113.031658 PMC3769317 23756428

[47] Guzman UH Martinez-Val A Ye Z Damoc E Arrey TN Pashkova A. Ultra-fast label-free quantification and comprehensive proteome coverage with narrow-window data-independent acquisition. Nat Biotechnol. (2024). doi: 10.1038/s41587-023-02099-7 PMC11631760 38302753

[48] Kawashima Y Watanabe E Umeyama T Nakajima D Hattori M Honda K. Optimization of data-independent acquisition mass spectrometry for deep and highly sensitive proteomic analysis. Int J Mol Sci. (2019) 20. doi: 10.3390/ijms20235932 PMC6928715 31779068

[49] Liebler DC Zimmerman LJ. Targeted quantitation of proteins by mass spectrometry. Biochemistry. (2013) 52:3797–806. doi: 10.1021/bi400110b PMC3674507 23517332

[50] Shao S Guo T Aebersold R. Mass spectrometry-based proteomic quest for diabetes biomarkers. Biochim Biophys Acta. (2015) 1854:519–27. doi: 10.1016/j.bbapap.2014.12.012 25556002

