# Supplementary material for: Inflammatory profile of eosinophils in asthma-COPD overlap and eosinophilic COPD: a multi-omics study
Source: Front Immunol. 2024 Oct 8;15:1445769. doi: 10.3389/fimmu.2024.1445769 (PMC11493663; doi:10.3389/fimmu.2024.1445769)
Supplement: Supplementary file 1 [file DataSheet1.pdf]

Supplementary Material

1 Supplementary Methods

1.1 RNA and Protein Extraction

Eosinophils were lysed with 0.5 mL of TRIzol Reagent (Thermo Fisher Scientific, Waltham, MA) and stored at −80 °C until use. Total RNAs and proteins were isolated from the lysate according to the manufacturer's protocols. In particular, the final protein pellet was washed twice with 0.8 mL of acetone, soaked in 4% SDS in 100 mM Tris-HCl, pH 8.0 for 24 hours, and then extracted proteins using a water bath-type sonicator (Bioruptor II; Cosmo Bio, Tokyo, Japan) on high power mode for 15 min in 30 s on/30 s off cycles. The protein extract was measured using a BCA Protein Assay Kit (Thermo Fisher Scientific, Waltham, MA, USA) and adjusted to 0.2 mg/ml with 4% SDS in 100 mM Tris-HCl, pH 8.0.

1.2 RNA-Seq Analysis

The concentration and the quality of the RNA were verified using a Qubit fluorometer (Thermo) and Agilent 2100 bioanalyzer, respectively. Purified total RNA was used for RNA library preparation, according to the instructions of the Quant Seq 3'mRNA-seq library preparation kit FWD of Illumina (Lexogen, Vienna, Austria). The RNA libraries were sequenced on an Illumina NextSeq 500 system with 75-nt-long reads. Prior to mapping, the adapter sequences were removed from the raw reads, and base trimming was performed from the 3' end of each read to remove bases with quality below Q10 up to a minimum length of 25 bp. Reads shorter than 25 bp were removed prior to further analysis. Each read was mapped to the human genome hg38 using Strand NGS (v4, Strand Life Sciences) and default settings (Minimum percent identity: 90%, Maximum percent gaps: 5 % and Minimum aligned read length: 25). After normalization of DESeq using default settings (threshold normalized counts: 1), pathway analyses were performed based on differentially expressed genes with a fold change ≥ 2 and p-value ≤ 0.05.

1.3 Proteome Analysis

100 µL of protein extract was treated with 20 mM tris (2-carboxyethyl phosphine) at 80 °C for 10 min and subjected to alkylation with 35 mM iodoacetamide in the dark at room temperature for 30 min while being protected from light. The alkylated sample was subjected to clean up and digestion with SP3 method (1, 2) with minor modifications. Briefly, two types of Sera-Mag SpeedBead carboxylate-modified magnetic particles (hydrophilic particles, CAT# 45152105050250; hydrophobic particles, CAT# 65152105050250; Cytiva, Marlborough, MA, USA) were used. These beads were combined at a 1:1 (v/v) ratio, washed twice with distilled water, and reconstituted in distilled water at a concentration of 10 µg solids/µL. 20 µL of reconstituted beads was then added to the alkylated protein sample followed by 99.5% ethanol to bring the final concentration to 75% ethanol, with mixing for 20 min. The supernatant was discarded, and the beads were washed twice with 80% ethanol. The beads were then resuspended in 100 µL of 50 mM Tris-HCl (pH 8.0) with 1 µg of trypsin/Lys-C Mix (CAT# V5072, Promega, Madison, WI, USA) and mixed gently at 37 °C for 14 h to digest proteins. The digested sample was acidified with 20 µL of 5% trifluoroacetic acid (TFA) and then sonicated using Bioruptor II. The sample was desalted using a SDB-STAGE tip

(CAT# 7820-11200, GL Sciences, Tokyo, Japan) according to the manufacturer's protocol, followed by drying in a centrifugal evaporator (miVac Duo concentrator, Genevac, Ipswich, UK) and redissolving in 2% ACN in 0.1% TFA. The peptide concentration in the redissolved sample was determined using a Lunatic instrument (Unchained Labs, Pleasanton, CA, USA) and transferred to a hydrophilic MS vial (CAT# 11-19-1021-10; AMR, Tokyo, Japan).

The digested peptides were directly injected onto an Aurora column (C18, 75 μm ID, 25 cm length, 1.6 μm beads, IonOpticks, Victoria, Australia) at 60°C and then separated with a 120-min gradient (A = 0.1% formic acid in water, B = 0.1% formic acid in 80% ACN) consisting of 0 min 5% B, 120 min 60% B at a flow rate of 150 nL/min using an UltiMate 3000 RSLCnano LC system. The peptides eluted from the column were analyzed by overlapping window DIA-MS (3, 4) using an Orbitrap Exploris 480 with an InSpIon system (5). MS1 spectra were collected in the range of *m/z* 495–745 at a 15,000 resolution to set an AGC targets of 3×10^6 and a maximum injection time of "Auto". MS2 spectra were collected at *m/z* 200-1,800 at a 60,000 resolution to set an AGC targets of 3×10^6, a maximum injection time of "Auto", and stepped normalized collision energies of 22, 26 and 30%. The isolation width for MS2 was set to 4 Da, overlapping window patterns at m/z 500–740 were used for window placements optimized via Scaffold DIA v3.2.1 (Proteome Software, Portland, OR, USA).

The MS files were searched against human spectral libraries using Scaffold DIA v3.2.1. The spectral library was generated from the human protein sequence database (Proteome id UP000005640, reviewed, canonical, 20381 entries, downloaded on March, 2021) by Prosit (6, 7). The Scaffold DIA search parameters were as follows: experimental data search enzyme, trypsin; maximum missed cleavage sites, 1; precursor mass tolerance, 10-ppm; fragment mass tolerance, 10-ppm; static modification, and cysteine carbamidomethylation. The protein identification threshold was set at < 1% for both peptide and protein false discovery rates. Peptide quantification was calculated by EncyclopeDIA algorithm (8) in Scaffold DIA. For each peptide, the four highest quality fragment ions were selected for quantitation. Protein quantification was estimated from the summed peptide quantification and normalized by according to the median protein quantification value.

The quantified protein data were transformed log2 (protein intensities) and filtered so that for each protein, at least one group contained a minimum of 70% valid values. The remaining missing values were imputed by random numbers drawn from a normal distribution (width, 0.3; downshift, 1.8) rather than fixed values to reduce statistical bias in Perseus v1.6.15.0 (9). The hierarchical clustering analysis was performed on the significant difference proteins with $p < 0.05$ (one-way ANOVA test), and clusters with downregulate against HS were extracted. Gene ontology enrichment analysis for the extracted proteins was performed using the CluGO v2.5.9 plugin (10) within the Cytoscape software v3.8.2 (11). The significance criterion is p-value < 0.05. The results were subsequently visualized, facilitating the clustering of each identified term.

1.4 Targeted Liquid Chromatography (LC)-Tandem Mass Spectrometry (MS/MS)-Based Lipidomics

Human eosinophils, isolated as detailed above, were suspended in Hank's buffered salt solution at 0.2×10^6 cells/mL and stimulated at 37°C for 30 min with 2 μM A23187. The incubation was stopped by centrifugation at 4°C for 5 min. The stimulated cell supernatant was then diluted in same volume of methanol and applied to MonoSpin C18-AX columns (GL Sciences, Inc., Tokyo, Japan), an anion-exchange cartridge with a hydrophobic retension character, for solid-phase extraction. A deuterated internal standard consisting of 1 ng of LTB_4-d4, LTD_4-d5, PGE_2-d4, and 15-HETE-d8 (Cayman

Chemical, Ann Arbor, MI) was added to the cell supernatants before extraction. We used a triple quadrupole linear ion trap mass spectrometer (QTRAP 5500; AB Sciex, Foster City, CA) equipped with a 1.7-µm, 1.0 × 150 mm Acquity UPLC™ BEH C18 column (Waters Corp., Milford, MA). MS/MS analyses were performed in negative ion mode, and the eicosanoids and docosanoids were identified and quantified by multiple reaction monitoring. Calibration curves were obtained over a 1–1000-pg range. LC retention times for each compound were determined with corresponding synthetic standards.

1.5 Quantitative Reverse Transcription (RT)-PCR

Total RNA was extracted from human eosinophils isolated as specified above using the RNeasy® Mini Kit (Qiagen, Hilden, Germany). The transcripts were quantified by RT-PCR, using the High Capacity RNA-to-cDNA Master Mix (Applied Biosystems, Foster City, CA), followed by quantitative PCR amplification with the CyberGreen method on an ABI PRISM® 7500 (Applied Biosystems, Foster City, CA). Primer sequences were summarized in supplementary table 1. The comparative threshold cycle method was validated and used to interpret the results. At least four samples from different donors were used in this analysis to ensure the reproducibility of the results.

1.6 Flow Cytometric Analysis of Cell Surface Markers

After the stimulation with the cytokines for cell culture for 72 h, eosinophils were labeled with the monoclonal antibodies for overnight at 4°C. The antibodies used in this study are summarized in supplementary table 2. Cell viability was evaluated by using propidium iodide (PI). The expression of these markers on PI- viable cells was analyzed using a FACS Gallios flow cytometer (Beckman Coulter, Inc., Miami, FL., USA) or CytoFLEX S (Beckman Coulter, Inc., Miami, FL., USA). All measurements were normalized to the value derived from the corresponding control isotype antibody. All data was analyzed by FlowJo software (v10.7.1). At least three samples from different donors were used in this analysis to ensure the reproducibility of the results.

1.7 References

1. Hughes CS, Moggridge S, Müller T, Sorensen PH, Morin GB, Krijgsveld J. Single-pot, solid-phase-enhanced sample preparation for proteomics experiments. Nat Protoc. 2019;14(1):68-85.
2. Hughes CS, Foehr S, Garfield DA, Furlong EE, Steinmetz LM, Krijgsveld J. Ultrasensitive proteome analysis using paramagnetic bead technology. Mol Syst Biol. 2014;10(10):757.
3. Amodei D, Egertson J, MacLean BX, Johnson R, Merrihew GE, Keller A, et al. Improving Precursor Selectivity in Data-Independent Acquisition Using Overlapping Windows. J Am Soc Mass Spectrom. 2019;30(4):669-84.
4. Kawashima Y, Nagai H, Konno R, Ishikawa M, Nakajima D, Sato H, et al. Single-Shot 10K Proteome Approach: Over 10,000 Protein Identifications by Data-Independent Acquisition-Based Single-Shot Proteomics with Ion Mobility Spectrometry. J Proteome Res. 2022;21(6):1418-27.
5. Kawashima Y, Ishikawa M, Konno R, Nakajima D, Ohara O. Development of a Simple and Stable NanoESI Spray System Using Suction Wind from the MS Inlet. J Proteome Res. 2023;22(5):1564-9.
6. Gessulat S, Schmidt T, Zolg DP, Samaras P, Schnatbaum K, Zerweck J, et al. Prosit: proteome-wide prediction of peptide tandem mass spectra by deep learning. Nat Methods. 2019;16(6):509-18.
7. Searle BC, Swearingen KE, Barnes CA, Schmidt T, Gessulat S, Küster B, et al. Generating high quality libraries for DIA MS with empirically corrected peptide predictions. Nat Commun. 2020;11(1):1548.
8. Searle BC, Pino LK, Egertson JD, Ting YS, Lawrence RT, MacLean BX, Villén J, MacCoss MJ.

Chromatogram libraries improve peptide detection and quantification by data independent acquisition mass spectrometry. Nat Commun. 2018;9(1):5128.
9. Tyanova S, Temu T, Sinitcyn P, Carlson A, Hein MY, Geiger T, et al. The Perseus computational platform for comprehensive analysis of (prote)omics data. Nat Methods. 2016;13(9):731-40.
10. Bindea G, Mlecnik B, Hackl H, Charoentong P, Tosolini M, Kirilovsky A, et al. ClueGO: a Cytoscape plug-in to decipher functionally grouped gene ontology and pathway annotation networks. Bioinformatics. 2009;25(8):1091-3.
11. Shannon P, Markiel A, Ozier O, Baliga NS, Wang JT, Ramage D, et al. Cytoscape: a software environment for integrated models of biomolecular interaction networks. Genome Res. 2003;13(11):2498-504.

2 Supplementary Table

Supplementary Table 1. Subject demographics by experimental analysis

	HP (N=5)	Non-eCOPD (N=5)	ACO/eCOPD (N=6)	Overall *P* value*	Pairwise *P* value# Non-eCOPD vs ACO/eCOPD
Age, y, Mean ± SD	32.2 ± 3.96	72.2 ± 4.14	69.8 ± 4.35	<0.01	0.38
Male sex, n (%)	5 (100)	5 (100)	6 (100)	N/A	>0.99
Clinical diagnosis of asthma, n (%)	0 (0)	0 (0)	4 (66.7)	0.01	0.06
Age at appearance of respiratory symptoms, y, ±SD	N/A	61.2 ± 3.27	51.8 ± 14.80	N/A	0.20
At least 10 pack-years of tabacco smoking, n (%)	0 (0)	5 (100)	6 (100)	<0.01	>0.99
Brinkman Index, Mean ± SD	0	1554 ± 757	1138 ± 636	<0.01	0.35
Inhaled steroid use, n (%)	0 (0)	0 (0)	5 (83.3)	<0.01	0.02
Leukotriene receptor antagonist use, n (%)	0 (0)	0 (0)	3 (50.0)	0.046	0.18
Blood eosinophil count (/µl)	N/A	91.1 ± 36.3	355.9 ± 236.6	N/A	0.04
Blood eosinophil proportion (%)	N/A	1.72 ± 0.66	5.67 ± 2.41	N/A	0.01
Blood neutrophil count (/µl)	N/A	3590 ± 774	3830 ± 1657	N/A	0.77
Total serum IgE (U/ml)	N/A	136 ± 145	648 ± 1222	N/A	0.38
BDR of FEV1 ⩾200 mL and 12% from baseline values on 1 visit	N/A	0	2	N/A	0.45
pre BD FEV1 (L)	N/A	1.96 ± 0.71	2.09 ± 0.37	N/A	0.70
pre BD FEV1% (%)	N/A	47.57 ± 14.60	59.7 ± 5.746	N/A	0.09
pre BD %FEV1 (%)	N/A	59.89 ± 19.75	72.93 ± 13.40	N/A	0.22
post BD FEV1 (L)	N/A	2.018 ± 0.63	2.27 ± 0.29	N/A	0.40
post BD FEV1% (%)	N/A	49.5 ± 14.91	63.29 ± 5.05	N/A	0.06
post BD %FEV1 (%)	N/A	62.34 ± 17.84	79.1 ± 10.61	N/A	0.08
ΔFEV1 (ml/year)	N/A	-106.49 ± 280.7	+138.81 ± 91.27	N/A	0.01
FeNO (ppb)	N/A	24.6 ± 12.10	33.3 ± 15.25	N/A	0.33
LAA on HRCT, n (%)	N/A	5	3 (50)	N/A	0.18
Only diffuse bronchial wall thickening on HRCT, n (%)	N/A	0	3 (50)	N/A	0.18

Data are shown as No. of patients (%) or Mean ± SD.

Abbreviation: ACO, asthma-COPD overlap; BD, bronchodilator; BDR, bronchodilator reversibility; COPD, chronic obstructive pulmonary disease; FeNO, fractional exhaled nitric oxide; FEV1, forced expiratory volume in 1 second; HP, healthy participants; HRCT, high resolusion computed tomography; LAA, low-attenuation areas.

*Ordinary one-way ANOVA or Chi-Square test, #Unpaired t test or Fisher's exact test

Supplementary Table 2. Correlations between gene and protein expression levels of the representative molecules

Gene	*P* value	r
MX2	0.003	0.7143
FCER1G	0.006	0.6701
MAP3K1	0.13	0.3956
ALOX5	0.16	0.3682
STAT5B	0.16	0.3646
NFKB1A	0.22	-0.3207
IL5RA	0.27	-0.2903
IL1RL1	0.28	0.2847
PTGDR2	0.29	-0.2798
SELPLG	0.34	-0.2529
ADORA2A	0.34	-0.2535
STAT1	0.61	0.1383
SEMA4A	0.73	0.0931
NOD2	0.76	0.0812
CLC	0.79	0.0712
NFKB1E	0.8	-0.0693
ORMDL3	0.85	0.0517
LSS	0.87	0.0460

3 Graphical Abstract

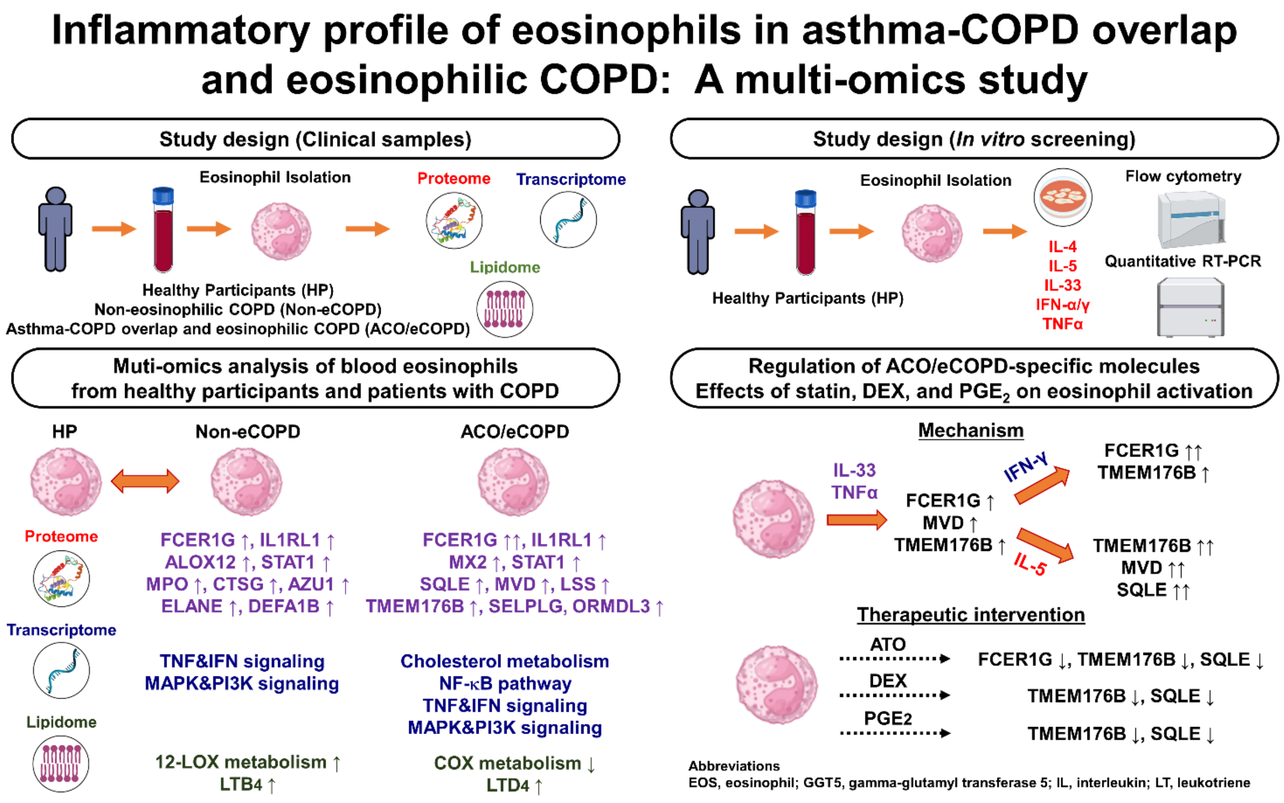

Graphical Abstract. Elevated blood eosinophil levels in patients with chronic obstructive pulmonary disease (COPD) with or without asthma are linked to increased exacerbations and the effectiveness of inhaled corticosteroid treatment. However, the role of eosinophils in the pathogenesis of COPD, especially asthma-COPD overlap (ACO), remains unclear. Multi-omics analysis revealed an inflammatory character associated with antiviral responses, cholesterol metabolism, and dysregulated cyclooxygenase metabolism of blood eosinophils in patients with ACO and eosinophilic COPD. Eosinophilic activation in COPD is associated with viral infection and a complex inflammatory milieu. Statin drugs and corticosteroids are optimal candidates to control these inflammatory changes.

4 Supplementary Figures

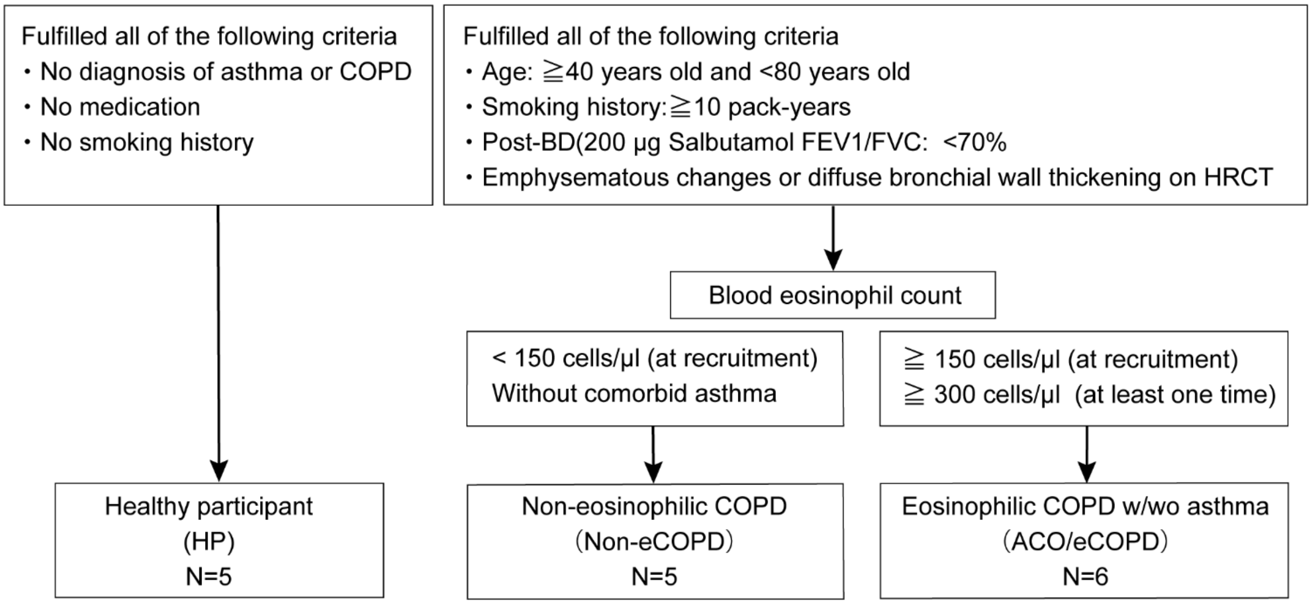

Supplementary Figure 1. Flow chart of the study population. The subjects in this study were composed of 5 healthy subjects with no history of asthma or chronic obstructive pulmonary disease (COPD) and 11 COPD patients aged ≤80 years and ≥40 years with <70% forced expiratory volume (FVC) % in one second (FEV1%) after bronchodilator (BD) administration, a smoking history of ≥10 pack-years, and emphysematous changes and/or diffuse bronchial wall thickening on chest computed tomography (CT). COPD patients were divided into two groups that were consisted of non-eosinophilic COPD (Non-eCOPD: 5 patients with blood eosinophils <150 cells/µL and without comorbid asthma) and asthma-COPD overlap (ACO) and/or eosinophilic COPD (eCOPD) (ACO/eCOPD: 6 patients with blood eosinophils ≥150 cells/µL and ≥300 cells/µL at any moment in the past).

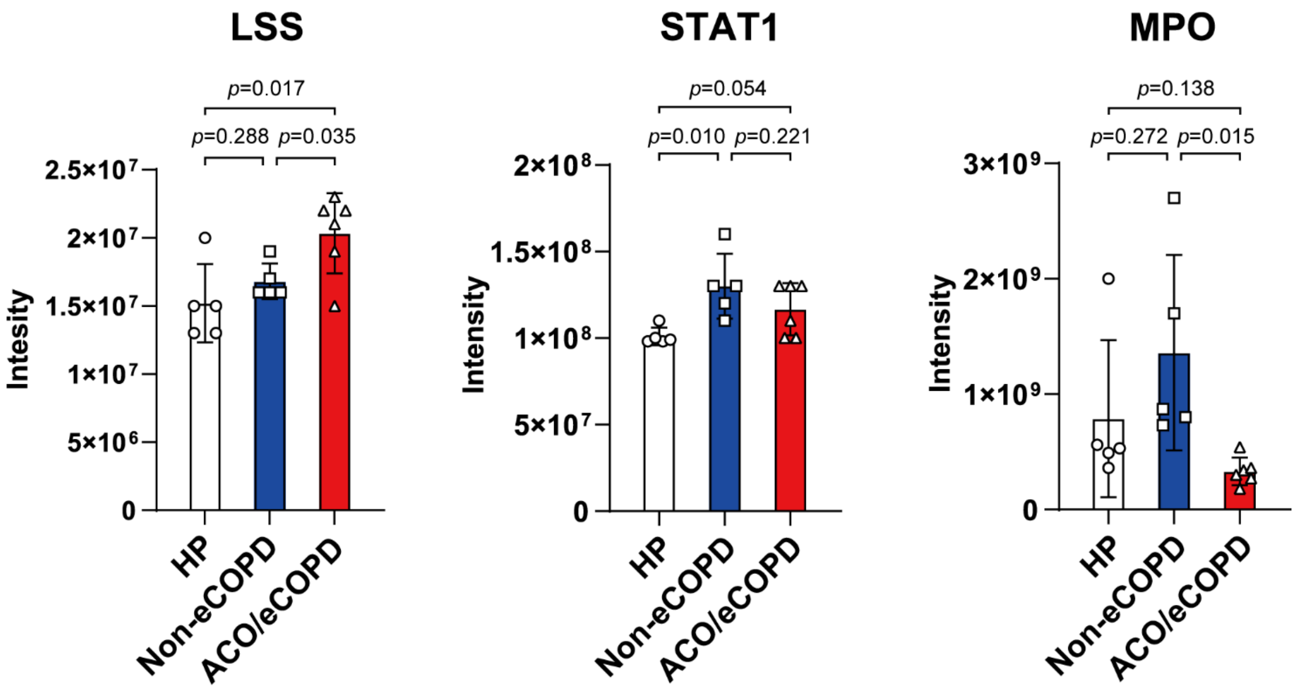

Supplementary Figure 2. Protein expressions of LSS, STAT1, and MPO in the data of proteomic analysis data. Cell lysates in phenol-guanidinium isothiocyanate reagent (P/GTC) were used for proteomic analysis. Protein expression levels of the representative molecules (LSS, STAT1, and MPO) with non-eosinophilic COPD (Non-eCOPD) or asthma-COPD overlap (ACO)/eosinophilic COPD (eCOPD)-specific upregulation were shown.

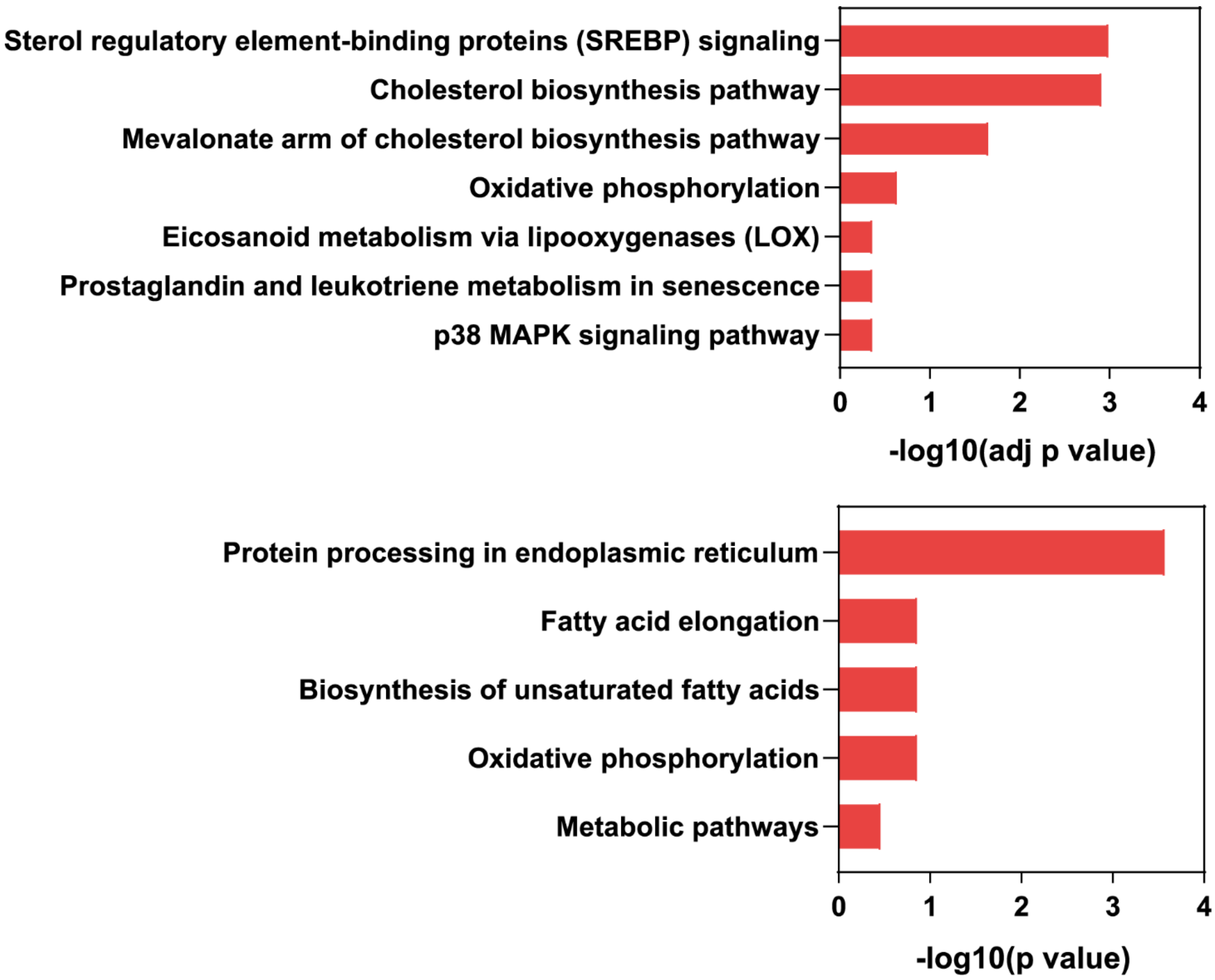

Supplementary Figure 3. Pathway analysis using the data of proteomic analysis. Cell lysates in phenol-guanidinium isothiocyanate reagent (P/GTC) were used for proteomic analysis. Pathway analysis of Wikipathways (Upper chart) and KEGG pathway (Lower chart) was performed using the data of the proteins classified into Cluster No. 3.

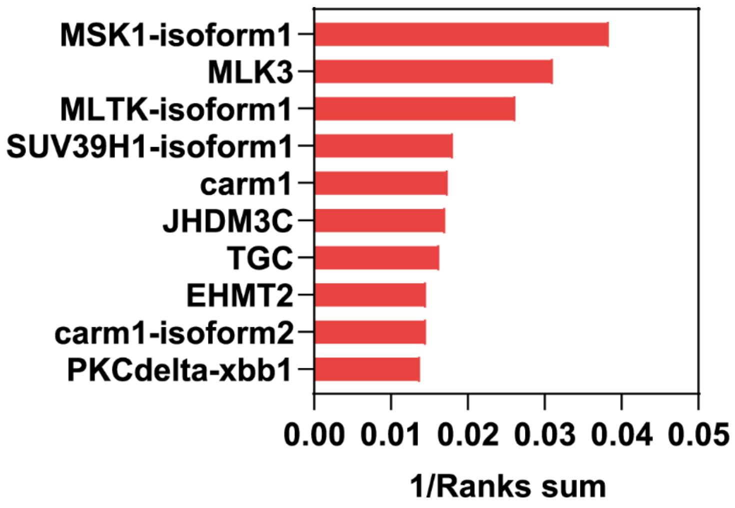

Supplementary Figure 4. Upstream analysis using the data of proteomic analysis. Cell lysates in phenol-guanidinium isothiocyanate reagent (P/GTC) were used for proteomic analysis. Upstream analysis was performed using the data of the proteins classified into Cluster No. 3.

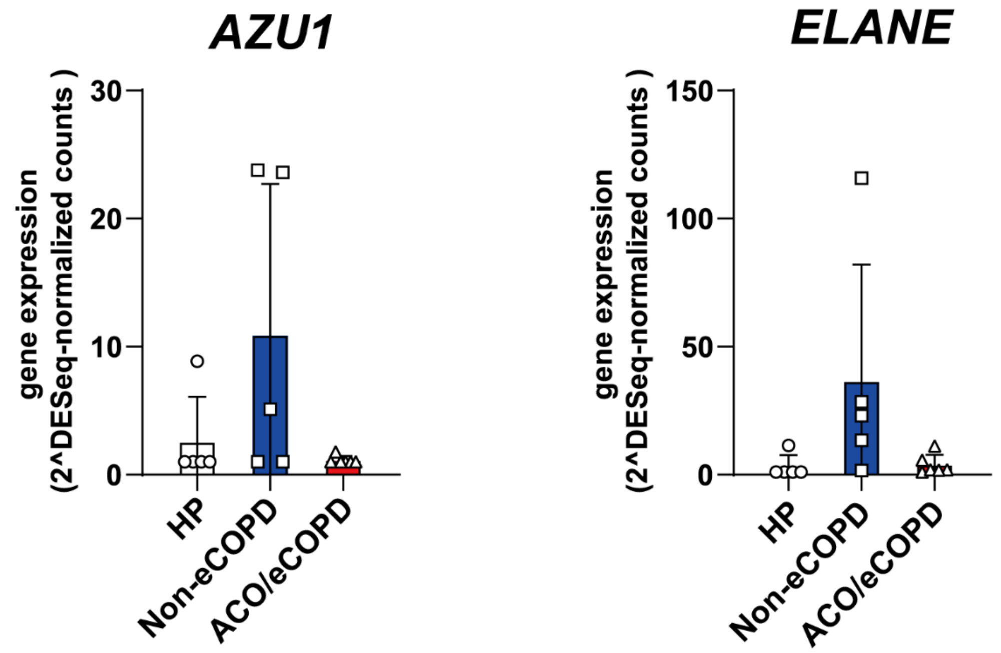

Supplementary Figure 5. Gene expressions of AZU1 and ELANE in the data of transcriptomic analysis data. Cell lysates in phenol-guanidinium isothiocyanate reagent (P/GTC) were used for transcriptomic analysis. Gene expression levels of the representative molecules (AZU1 and ELANE) with non-eosinophilic COPD-specific upregulation were shown.

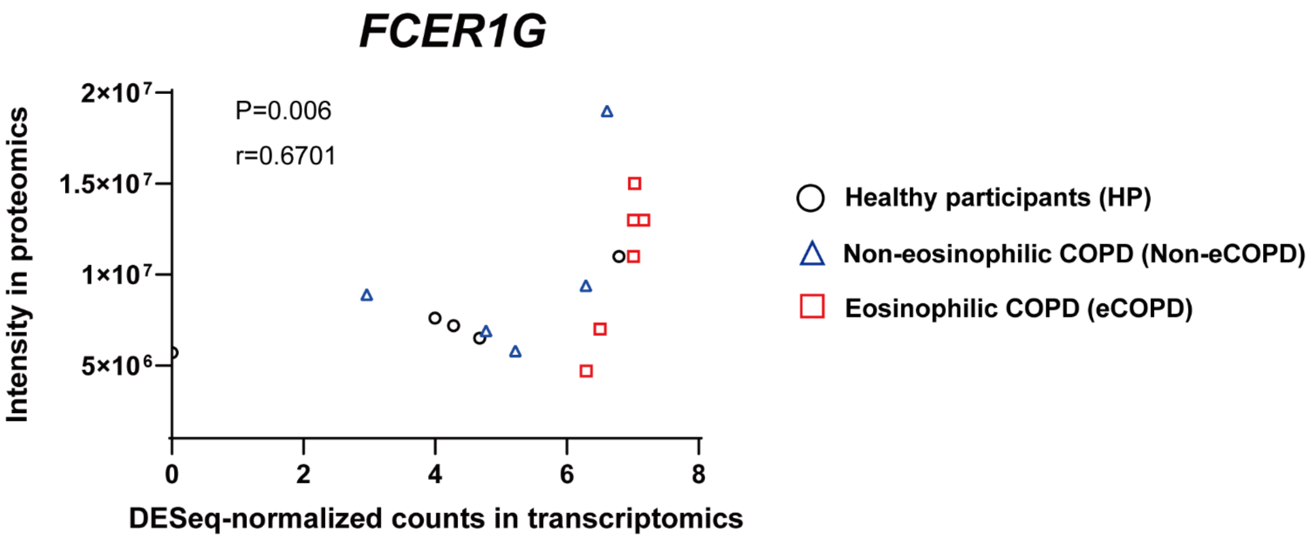

Supplementary Figure 6. Correlations between FCER1G gene and protein expression levels. Correlation analysis was conducted using data from both transcriptomic and proteomic analyses. A statistically significant correlation was observed between the gene and protein expression levels of FCER1G.

Data

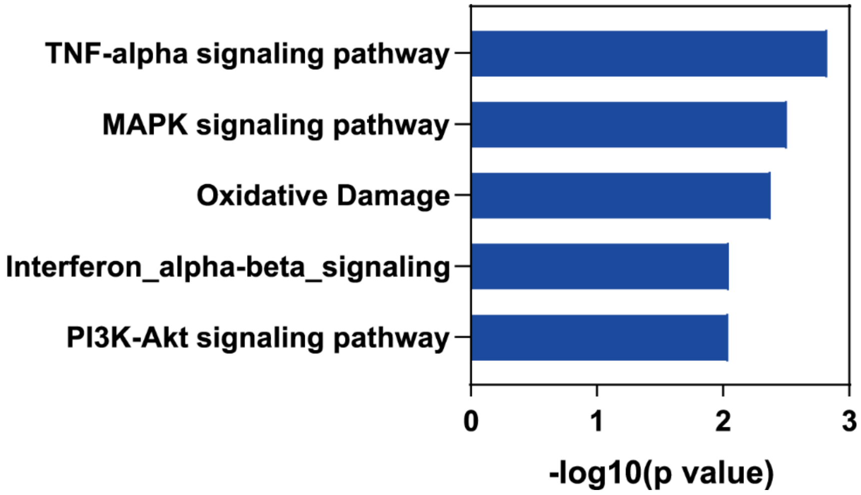

Supplementary Figure 7. Pathway analysis using the data of transcriptomic analysis data. Wikipathways analysis annotated keyword classification and functional enrichment for the upregulated molecules whose expressions were significantly higher in overall COPD than those in healthy subjects was performed.

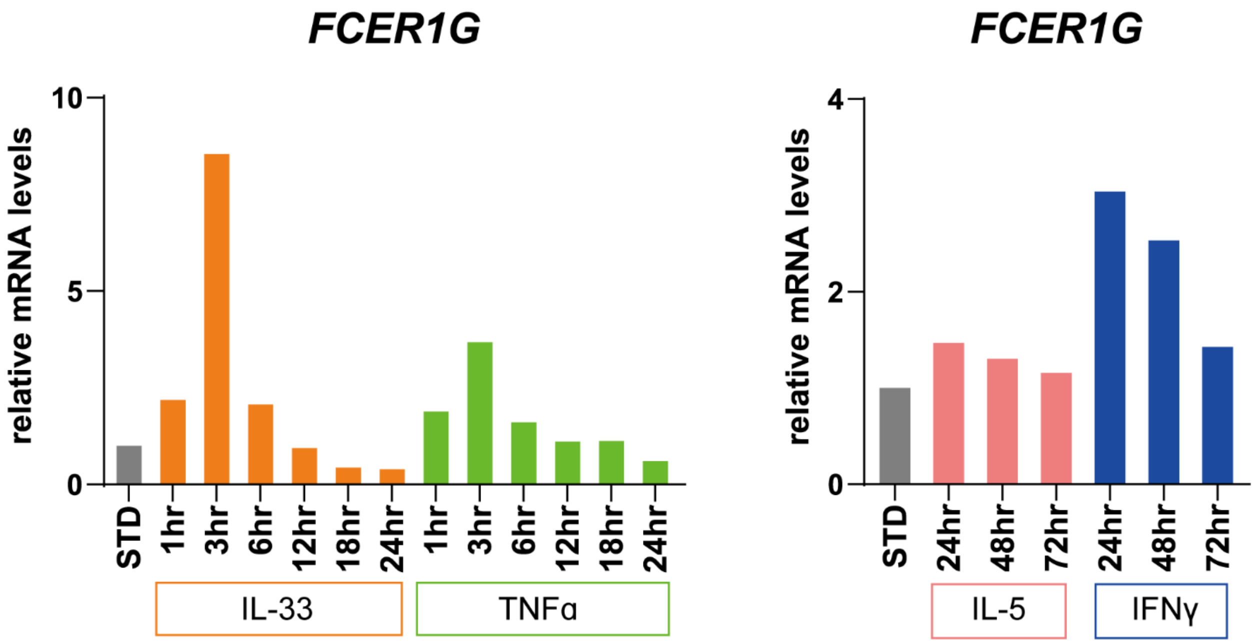

Supplementary Figure 8. Time course of the upregulation of mRNA expression of FCER1G in eosinophils upon stimulation with various cytokines. Human eosinophils were isolated from peripheral blood of healthy subjects and stimulated with 10 ng/mL various cytokines (IL-33, TNF-α, IL-5, and IFN-γ) for 1-24 h (IL-33 and TNF-α) or 24-72 h (IL-5 and IFN-γ). Gene expression levels of FCER1G were evaluated by RT-PCR.

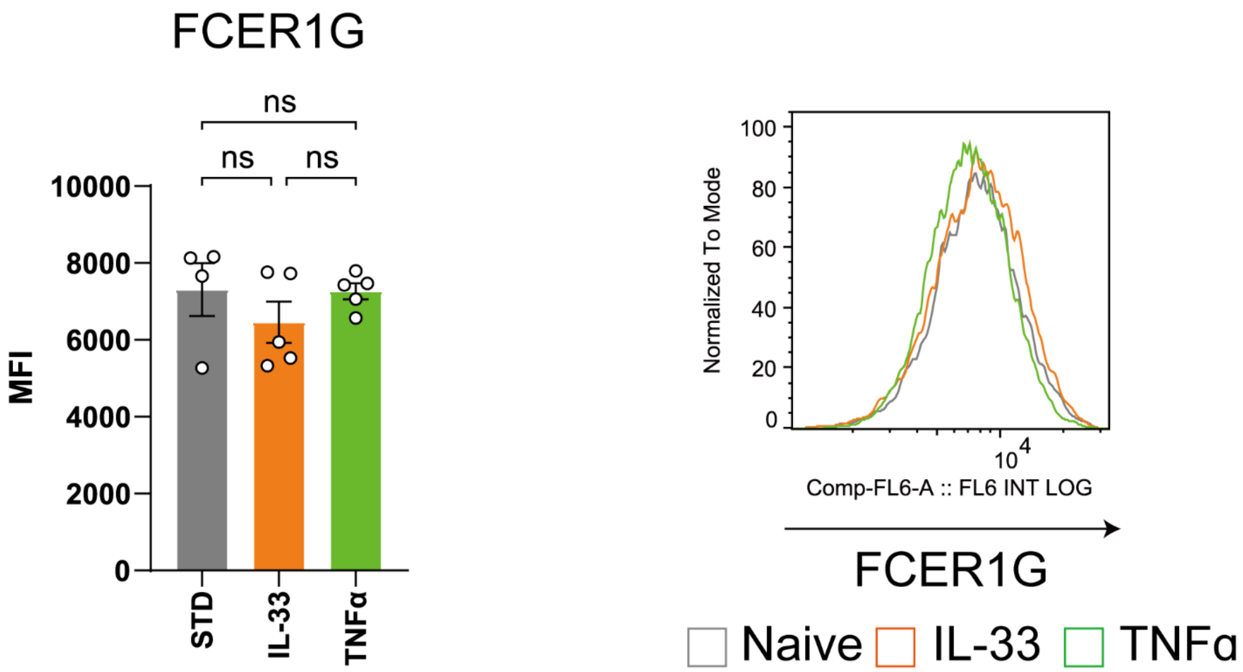

Supplementary Figure 9. Cell surface expressions of FCER1G on eosinophils stimulated with IL-33 or TNF-α. Human eosinophils were isolated from peripheral blood of healthy subjects and stimulated with or without 10 ng/mL IL-33 or TNF-α. Mean fluorescence intensity (left) and histogram (right) of FCER1G of the stimulated cells were evaluated using flowcytometry.

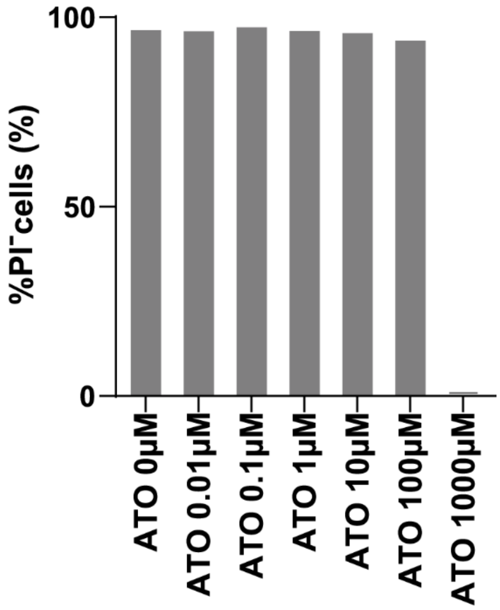

Supplementary Figure 10. Cytotoxicity of atorvastatin on eosinophils. Human eosinophils were isolated from peripheral blood of healthy subjects were treated with atorvastatin at the concentration of 0.01 μM -1 mM. Cell viability was evaluated to measure the ratio of propidium iodide (PI)-negative cells in eosinophils using flowcytometry.

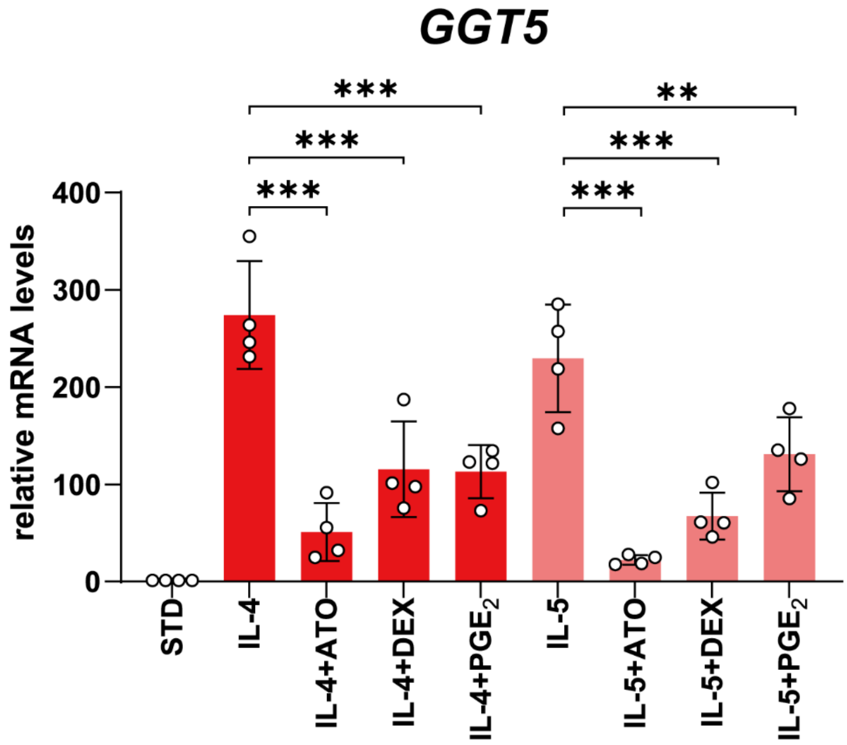

Supplementary Figure 11. Suppressive effects of atorvastatin, dexamethasone and prostaglandin E_2 on GGT5 mRNA expressions. Human eosinophils were isolated from peripheral blood of healthy subjects and stimulated with 10 ng/mL IL-4 or IL-5 for 3 h after pretreatment of atorvastatin (ATO), dexamethasone (DEX), and prostaglandin E_2 (PGE_2). Gene expression levels of GGT5 were evaluated by RT-PCR. Mean ± SEM, n=4-6 for each group. *P<0.05, **P<0.01, ***P<0.001. Data are representative of at least three independent experiments.
